# Molecular grafting towards high-fraction active nanodots implanted in N-doped carbon for sodium dual-ion batteries

**DOI:** 10.1093/nsr/nwaa178

**Published:** 2020-08-25

**Authors:** Sainan Mu, Qirong Liu, Pinit Kidkhunthod, Xiaolong Zhou, Wenlou Wang, Yongbing Tang

**Affiliations:** Functional Thin Films Research Center, Shenzhen Institutes of Advanced Technology, Chinese Academy of Sciences, Shenzhen 518055, China; Nano Science and Technology Institute, University of Science and Technology of China, Suzhou 215123, China; Functional Thin Films Research Center, Shenzhen Institutes of Advanced Technology, Chinese Academy of Sciences, Shenzhen 518055, China; Synchrotron Light Research Institute, Nakhon Ratchasima 30000, Thailand; Functional Thin Films Research Center, Shenzhen Institutes of Advanced Technology, Chinese Academy of Sciences, Shenzhen 518055, China; Nano Science and Technology Institute, University of Science and Technology of China, Suzhou 215123, China; Functional Thin Films Research Center, Shenzhen Institutes of Advanced Technology, Chinese Academy of Sciences, Shenzhen 518055, China; Key Laboratory of Advanced Materials Processing & Mold, Ministry of Education, Zhengzhou University, Zhengzhou 450002, China

**Keywords:** molecular grafting, high-fraction active material, tin pyrophosphate, N-doped carbon, sodium-based dual-ion batteries

## Abstract

Sodium-based dual-ion batteries (Na-DIBs) show a promising potential for large-scale energy storage applications due to the merits of environmental friendliness and low cost. However, Na-DIBs are generally subject to poor rate capability and cycling stability for the lack of suitable anodes to accommodate large Na^+^ ions. Herein, we propose a molecular grafting strategy to *in situ* synthesize tin pyrophosphate nanodots implanted in N-doped carbon matrix (SnP_2_O_7_@N-C), which exhibits a high fraction of active SnP_2_O_7_ up to 95.6 wt% and a low content of N-doped carbon (4.4 wt%) as the conductive framework. As a result, this anode delivers a high specific capacity ∼400 mAh g^−1^ at 0.1 A g^−1^, excellent rate capability up to 5.0 A g^−1^ and excellent cycling stability with a capacity retention of 92% after 1200 cycles under a current density of 1.5 A g^−1^. Further, pairing this anode with an environmentally friendly KS6 graphite cathode yields a SnP_2_O_7_@N-C||KS6 Na-DIB, exhibiting an excellent rate capability up to 30 C, good fast-charge/slow-discharge performance and long-term cycling life with a capacity retention of ∼96% after 1000 cycles at 20 C. This study provides a feasible strategy to develop high-performance anodes with high-fraction active materials for Na-based energy storage applications.

## Teaser

High-fraction active material (95.6 wt%) implanted in nitrogen-doped carbon matrix is designed for sodium-based dual-ion batteries with superior rate performance and long-term cycling life.

## INTRODUCTION

The limited reserve and uneven distribution of lithium resource promote the development of lithium-free energy storage systems based on abundant alkali and alkaline cations such as Na^+^ [1–9], K^+^ [10–15], Mg^2+^ [16–18], Ca^2+^ [19,20], Zn^2+^ [21–25], Al^3+^ [26–28], etc. Among them, owing to the high natural abundance of sodium resources and the similar electrochemical properties of Na^+^ to Li^+^, sodium-ion batteries (SIBs) are a potential alternative to lithium-ion batteries (LIBs) for large-scale power grids and intermittent energy storage systems [29–36]. On the other hand, dual-ion batteries (DIBs) have also attracted considerable attention due to their advantages of high working voltages, environmental benignity and low cost [37–42]. In this cell configuration, graphite materials are generally applied as both anode and cathode, cations and anions participate in the electrochemical redox reactions on anode and cathode, respectively [43–47]. Therefore, if the advantages of both SIBs and DIBs are combined, it is possible to develop high efficient, low-cost and environmentally friendly sodium-based DIBs (Na-DIBs) for large-scale energy storage applications.

However, unlike Li^+^ and K^+^ ions, it is difficult for traditional graphite materials to act as the anode for the intercalation of Na^+^ ions [48,49]. Further, the large ionic radius of Na^+^ (1.02 Å vs. 0.76 Å for Li^+^) results in sluggish reaction kinetics and large volume changes of the anode materials such as Sn [50,51], MoS_2_ [52–54], TiO_2_ [55] and FePO_4_ [56], and thus leads to poor rate capability and unsatisfied cycling stability [57–59]. Several approaches have been applied to improve the electrochemical performance of these anodes, including nanoscale modification and carbon-based composite construction [60–65], for example, carbon-based tin pyrophosphate (SnP_2_O_7_) composite with a high carbon content (16.8%) has been demonstrated to exhibit enhanced cycling stability for Na^+^-ion storage [66]. Although the carbon matrix can improve the electronic conductivity and provide a buffer framework for alleviating the volume expansion of these anodes, the excessive carbon content (commonly >15 wt%) would decrease the fraction of active material and thus reduce the energy density of batteries. Therefore, it is necessary to increase the fraction of active materials as high as possible and reduce the content of inactive carbon without compromising the conductivity of composite anodes, so that anodes can effectively deliver their specific capacities.

Herein, we propose a molecular grafting strategy to *in situ* implant SnP_2_O_7_ nanostructure in N-doped carbon (SnP_2_O_7_@N-C) as the anode for Na-DIBs. Such a strategy enables high-fraction (95.6 wt%) active materials to uniformly embed in the carbon matrix and to effectively prevent the exfoliation of active materials, while the N doping leads to high conductivity even at a low C content. It exhibits a high specific capacity of 400 mAh g^−1^ at 0.1 A g^−1^ and excellent cycling stability with a capacity retention of 92% after 1200 cycles under 1.5 A g^−1^. Consequently, pairing this anode with an environmentally friendly graphite cathode yields a SnP_2_O_7_@N-C||KS6 Na-DIB, which shows excellent rate performance up to 30 C, good fast-charge/slow-discharge ability and long-term cycling life with a capacity retention of 96.3% after 1000 cycles, showing a promising potential for Na-based energy storage devices.

## RESULTS AND DISCUSSION

**Figure 1.**
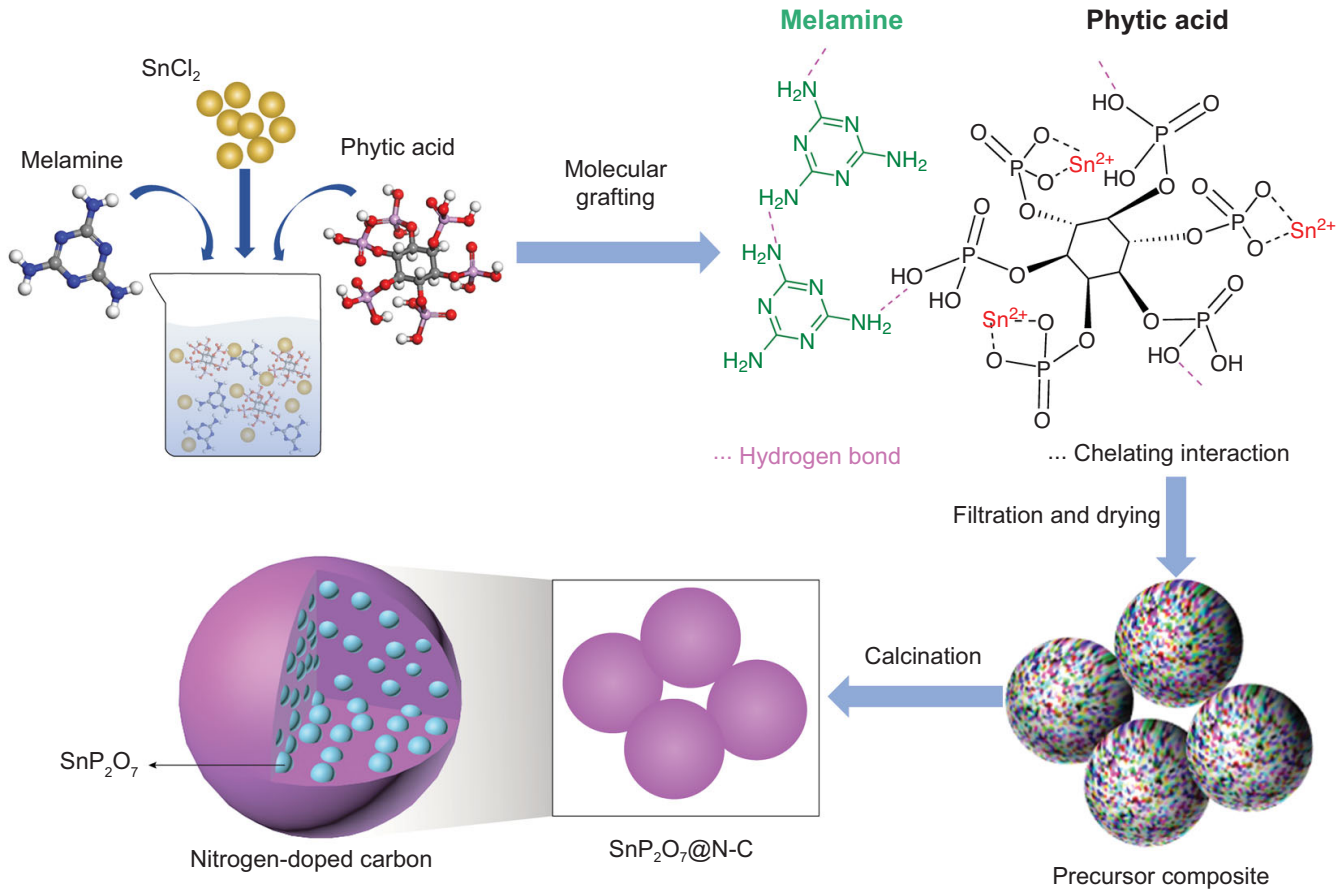
Schematic synthesis process of the SnP_2_O_7_@N-C composite.

Figure 1 schematically illustrates the synthesis procedure of SnP_2_O_7_@N-C via the molecular grafting method. Owing to the complexing interaction between radical groups (e.g. phosphate groups) and metal cations (e.g. tin ions) and the hydrogen bond between organic precursors, many precursor agents can molecularly graft into precursor composite with a three-dimensional framework, accompanied by a full mixing procedure. In this case, we chose phytic acid as the phosphorous source to strengthen the adhesion between active nanodots and carbon matrix due to sufficient O-C bonds and strong complexing ability of phosphate groups to tin cations. Simultaneously, the low atomic ratio of C to P can avoid residual carbon content in the formed composite. Besides, we chose the melamine as the N doping source because its high atomic ratio of N to C can result in a high concentration of nitrogen in the carbon matrix. After the filtration and drying processes, composite precursor nanoparticles were achieved. Finally, the SnP_2_O_7_/N-C nanoparticles were synthesized via calcining the precursor composite under an Ar atmosphere.

In order to investigate the molecular grafting process, Fourier transform infrared spectra (FTIR) measurements of precursors and their composites were carried out. The characteristic absorption peaks originating from phosphate radical (980 and 1140 cm^−1^), phosphate hydrogen radical (1631 cm^−1^) and stretching vibration of O-H (3329 cm^−1^) are observed in the FTIR spectrum of the phytic acid solution (Supplementary Fig. S1a). To confirm the complexing interaction between phosphate groups and tin cations, phytic acid and SnCl_2_ were applied to synthesize a precursor composite without the addition of melamine. Compared with that of phytic acid, the FTIR spectrum of the composite (Supplementary Fig. S1b) presents an obvious peak shift of phosphate to 1035 cm^−1^, which should be ascribed to the complexing interaction between phosphate groups and tin cations. For the pure melamine, some characteristic absorption peaks involving the out-of-plane ring bending vibration of triazine ring (810 cm^−1^), stretching vibration of C-N (1431 cm^−1^), stretching vibrations of triazine ring (1526 cm^−1^), scissoring vibration of NH_2_ (1626 cm^−1^) and stretching vibrations of NH_2_ (3100–3500 cm^−1^) were observed in its FTIR spectrum (Supplementary Fig. S1c). Once melamine had been added, its three typical absorption peaks at 773, 1440 and 1529 cm^−1^ were detected in the FTIR spectrum of the precursor composite (Supplementary Fig. S1d). The obvious peak shift of melamine at 810 to 773 cm^−1^ should be attributed to the formation of intermolecular hydrogen bonds between melamine and phosphate groups [67].

**Figure 2.**
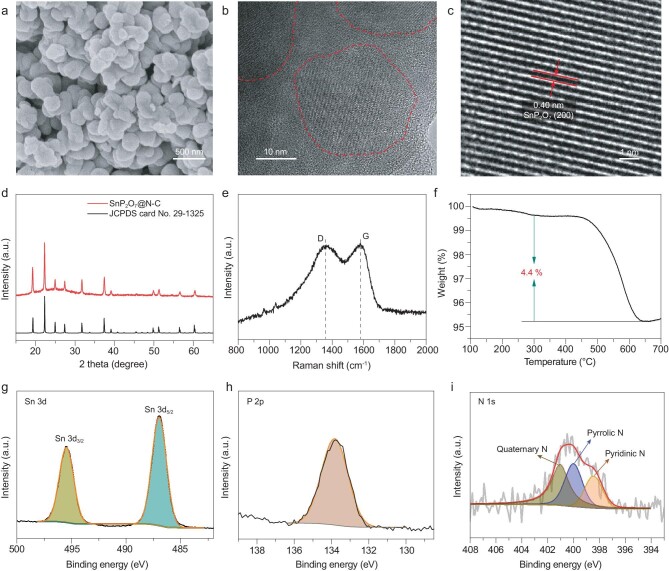
Morphology, microstructure and chemical components of as-synthesized SnP_2_O_7_@N-C. (a) SEM image, (b and c) HRTEM images, (d) XRD pattern, (e) Raman spectrum and (f) TGA analysis of as-synthesized SnP_2_O_7_@N-C. High-resolution XPS spectra of Sn 3d (g), P 2p (h) and N 1s (i).

Compared with the precursor composite (Supplementary Fig. S2), the synthesized SnP_2_O_7_@N-C composite features a stable morphology without structural collapse after calcination treatment (Fig. 2a and Supplementary Fig. S3a–c), and is comprised of nanoparticles with an average size of 200 nm. The selected area electron diffraction (SAED) pattern (Supplementary Fig. S3d) indicates that the SnP_2_O_7_@N-C sample has a well-crystallized structure. Further characterizations via high-resolution transmission electron microscopy (HRTEM) images (Fig. 2b) detect that several crystalline nanodots are uniformly implanted in the amorphous carbon matrix. Figure 2c and Supplementary Fig. S3e show obvious lattice fringes with an interplanar spacing of 0.40 nm, matching well with the (200) plane of cubic-phase SnP_2_O_7_. X-ray diffraction (XRD) pattern and Raman spectrum were carried out to provide more structural information. As observed in Fig. 2d, all sharp diffraction peaks can be indexed to cubic-phase SnP_2_O_7_ (JCPDS Card No. 29-1352), in accordance with the HRTEM observations. In contrast, those samples calcined at 500°C and 700°C (Supplementary Fig. S4) do not present similar characteristic diffraction peaks of SnP_2_O_7_. Note that a bump peak located at ∼26° should originate from the amorphous carbon matrix. Its amorphous feature is also confirmed by the Raman spectrum (Fig. 2e), where two characteristic peaks of carbon situated at ∼1360 and ∼1585 cm^−1^ can be observed. Both peaks are individually attributed to D band of disordered carbon and G band of graphitic carbon. The ratio of *I*_D_ to *I*_G_ approximates 1.0, implying the carbon matrix's dominant defective and disordered nature. Further thermogravimetric analysis (TGA) measurement (Fig. 2f) indicates that the fractions of SnP_2_O_7_ nanodots and N-doped carbon are 95.6 wt% and 4.4 wt%, respectively, which is the highest fraction of active material among previously reported Sn-based compound/carbon composites [66,68–70]. Both the XRD pattern (Supplementary Fig. S5a) and the Raman spectrum (Supplementary Fig. S5b) after the TGA test show the absence of carbon characteristic peaks, indicating the complete decomposition of the carbon component in the TGA test. Similarly, the TGA analysis of SnP_2_O_7_@C (Supplementary Fig. S6) shows that the carbon content of the SnP_2_O_7_@C composite is ∼3.7%, close to that of SnP_2_O_7_@N-C (∼4.4%), which suggests that the addition of melamine slightly increases the carbon content, ascribable to its high atomic ratio of N to C. The nitrogen adsorption/desorption isotherm of SnP_2_O_7_@N-C (Supplementary Fig. S7) reveals that its Brunauer-Emmert-Teller (BET) specific surface area is ∼9.0 m^2^ g^−1^.

The chemical components of the SnP_2_O_7_@N-C sample were analyzed by X-ray photoelectron spectroscopy (XPS). As shown in Supplementary Fig. S8a, the survey XPS spectrum suggests the existence of O, P, Sn, C and N elements in the sample, consistent with the energy dispersive X-ray spectroscopy (EDX) elemental mappings where these elements uniformly distribute in the SnP_2_O_7_@N-C composite (Supplementary Fig. S9). High-resolution Sn 3d XPS spectrum (Fig. 2g) presents a pair of characteristic peaks at 495.4 and 487.0 eV, corresponding to Sn 3d_3/2_ and Sn 3d_5/2_ of Sn^4+^ in SnP_2_O_7_, respectively. Besides, only one peak at 134.0 eV referring to the P 2p can be observed (Fig. 2h), indicating a complete transformation of P source to SnP_2_O_7_ without P doping. The deconvoluted O 1s spectrum (Supplementary Fig. S8b) includes two peaks. The dominant peak is assigned to the SnP_2_O_7_, and another involves O-C bonding. Moreover, the high-resolution C 1s spectrum (Supplementary Fig. S8c) can be fitted into three peaks at 284.6, 285.5 and 286.5 eV, individually originating from C-C, C-N and C-O, exhibiting that the carbon matrix is doped with dominant nitrogen and slight oxygen [54]. The high-resolution N 1s spectrum (Fig. 2i) shows the existence of pyridinic N (398.5 eV), pyrrolic N (400.0 eV) and quaternary N (401.1 eV) [71]. Such structural and chemical features confirm the homogeneous implantation of SnP_2_O_7_ nanodots in the N-doped carbon matrix, which is expected to optimize its charge transfer kinetics and electrochemical stability for SIBs.

**Figure 3.**
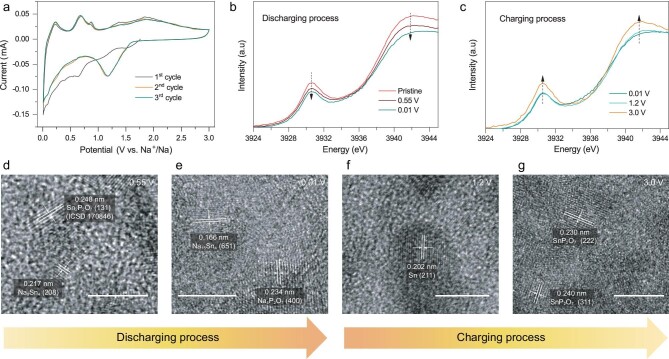
Studies on the working mechanism of SnP_2_O_7_@N-C in the sodium-based half-cell. (a) First three CV curves at a sweep rate of 0.1 mV s^−1^. (b and c) Sn L_3_-edge XANES spectra of the SnP_2_O_7_@N-C anode during discharging (b) and charging (c) processes. (d–g) HRTEM images of SnP_2_O_7_@N-C anode at different discharging states of (d) 0.55 V and (e) 0.01 V, and different charging states of (f) 1.2 V and (g) 3.0 V. Scale bars: 5 nm.

We firstly carried out the cyclic voltammogram (CV) measurement to investigate the Na^+^-storage behavior of the SnP_2_O_7_@N-C anode. Figure 3a exhibits the first three CV curves at 0.1 mV s^−1^ in the potential range of 0.01–3.0 V. In the first sodiation process, there are multiple peaks situated at 1.55, 1.10, 0.58, 0.39 and 0.07 V. According to previous reports, the Na-Sn alloying reactions occurred at potentials below 0.9 V [70,72]. Thus, the first two peaks should involve the conversion process of SnP_2_O_7_ to metallic Sn, and the others stem from the Na–Sn alloying reactions [70,71]. In the following cycles, only a broad and strong peak at 1.18 V is observed for the conversion reaction. However, the desodiation processes in different cycles always exhibit five peaks at 0.23, 0.69, 0.86, 1.38 and 1.84 V. Such behavior suggests the conversion reaction probably refers to a two-step reduction/oxidation reaction of Sn^4+^/Sn^2+^ and Sn^2+^/Sn^0^, and the Na–Sn alloying/dealloying reaction is also associated with a multi-step reaction process.

To further get insight into its Na^+^-ion storage mechanism, the sodiation/desodiation process of the SnP_2_O_7_@N-C anode was detected with synchrotron X-ray absorption near edge structure (XANES) spectra of the Sn L_3_-edge (Fig. 3b and c) at different charging/discharging states (Supplementary Fig. S10), where two peaks are assigned to the 2p_3/2_–5s_1/2_ transition [73]. As observed in Fig. 3b, the intensities of the characteristic peaks decrease as the discharging process proceeds, associated with the transformation of Sn^4+^ to Sn^0^ [73,74]. A reverse evolution of the peak intensities is obviously observed during the charging process (Fig. 3c), demonstrating the good reversibility of Sn state during cycling. Note that the slight difference of Sn L_3_-edge XANES spectra at 0.01 V and 1.2 V should be ascribed to the de-alloying reactions of the sample without obvious variation in the valence state of Sn element.

Further, HRTEM characterizations at different charging/discharging states were performed to verify its sodiation/desodiation mechanism. For the pristine sample, an interplanar spacing of 0.398 nm is clearly distinguished (Supplementary Fig. S11a), which corresponds to (200) plane of the SnP_2_O_7_. When the sodiation process proceeds until 0.55 V, there are some lattice fringes with lattice spacing of 0.248 and 0.217 nm (Fig. 3d), which match well with (131) plane of Sn_2_P_2_O_7_ (ICSD No. 170846) and (208) plane of Na_9_Sn_4_ (PDF No. 31-1326), respectively. And the fully sodiated state clearly contains Na_4_P_2_O_7_ and Na_15_Sn_4_ two crystal phases (Fig. 3e), further confirming that the sodiation process of SnP_2_O_7_@N-C anode involves both conversion and alloying reactions. Conversely, as the desodiation process is conducted to 1.2 V, the presence of metallic Sn is verified by the HRTEM image in Fig. 3f. The completed desodiation process at 3.0 V is accompanied by the formation of SnP_2_O_7_ (Fig. 3g), indicating a good sodiation/desodiation reversibility of SnP_2_O_7_. It is also noteworthy that, differently from the reported results [66], there are some lattice fringes with an interplanar spacing of 0.304 nm (Supplementary Fig. S11b), corresponding to the (−131) plane of P-1 Sn_2_P_2_O_7_ (ICSD No. 170846), which implies the presence of Sn_2_P_2_O_7_ during the desodiation process. Therefore, the HRTEM result is greatly consistent with the analyses of CV result during sodiation/desodiation processes.

**Figure 4.**
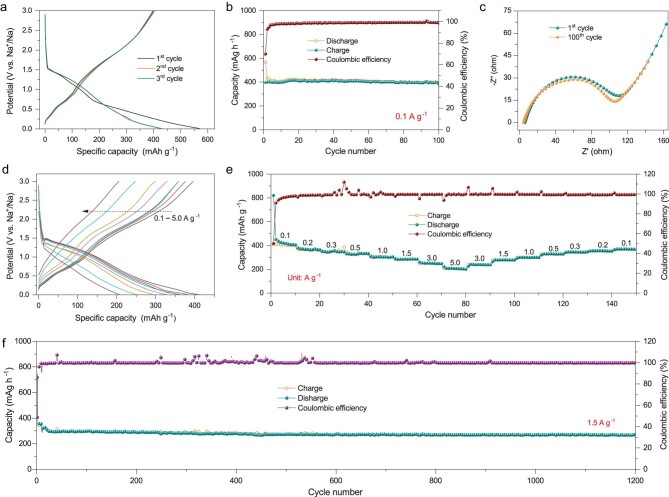
Electrochemical performances of the SnP_2_O_7_@N-C anode in sodium-based half-cells. (a) Galvanostatic charge–discharge profiles and (b) the corresponding cycling performance at a current density of 0.1 A g^−1^. (c) Nyquist plots of the SnP_2_O_7_@N-C anode before and after 100 cycles. (d) Galvanostatic charge–discharge profiles measured at different current densities and (e) the corresponding rate capability. (f) Long-term cycling stability at 1.5 A g^−1^.

The electrochemical properties of the SnP_2_O_7_@N-C anode were evaluated in a coin-type half-cell. As observed in Fig. 4a, an abnormal shape of the galvanostatic charge–discharge profile at the first cycle is attributed to the incompletely reversible sodiation process of SnP_2_O_7_ and the formation of solid-electrolyte interphase (SEI) layer [54,75]. Although a pulverization phenomenon of SnP_2_O_7_ is observed after the first sodiation/desodiation process (Supplementary Fig. S12), there is a stable shape of galvanostatic charge–discharge profiles after the first cycle. It shows a specific discharge capacity of ∼400 mAh g^−1^ at 0.1 A g^−1^ with a Coulombic efficiency of *∼*100% (Fig. 4b), indicating a good stability during the following sodiation/desodiation processes. Further, the EDX mappings of the anode at fully discharged state (Supplementary Fig. S13) verify uniform distributions of O, P, Sn, Na, C and N elements, implying a homogeneous sodiation reaction during discharging process. Such robust charging/discharging behavior was also confirmed by the electrochemical impedance spectroscopy (EIS, Fig. 4c). No obvious variation in its EIS spectra is observed before and after 100 cycles, ascribable to the strong adhesion between SnP_2_O_7_ nanodots and N-doped carbon matrix.

Figure 4d and e present the rate performance of SnP_2_O_7_@N-C anode at current densities from 0.1 to 5.0 A g^−1^. It exhibits specific capacities of 400, 381, 354, 335, 305, 295, 261 and 210 mAh g^−1^ at current densities of 0.1, 0.2, 0.3, 0.5, 1.0, 1.5, 3.0 and 5.0 A g^−1^, respectively. The specific capacities are recoverable as the current density is returned to 0.1 A g^−1^. It should be noted that the rate capability of the SnP_2_O_7_@N-C anode is much better than that of pure SnP_2_O_7_ (58 mAh g^−1^ at 1.5 A g^−1^), SnP_2_O_7_@C without N doping (176 mAh g^−1^ at 1.5 A g^−1^) (Supplementary Fig. S14) and previously reported SnP_2_O_7_ composite with 16.8 wt% carbon nanosheets [66]. The excellent rate capability can be attributed to that N doping enhances the conductivity of carbon framework and facilitates diffusion kinetics of Na^+^ ions [71,76], and the active nanodots shorten the diffusion path of Na^+^ ions [77,78]. Figure 4f and Supplementary Fig. S15 show the composite anode's cycling performance under a current density of 1.5 A g^−1^, exhibiting excellent cycling stability with a capacity retention ∼92% after 1200 cycles and the corresponding Coulombic efficiency close to 100%. In contrast, much lower capacity retentions of ∼79% and ∼49% are obtained for SnP_2_O_7_@C and pure SnP_2_O_7_ after 400 cycles (Supplementary Fig. S16), respectively. Among the reported Sn-based compound/carbon composite anodes for SIBs (Supplementary Table S1), the SnP_2_O_7_@N-C with the lowest carbon content delivers a competitive specific capacity and superior cycling performance.

**Figure 5.**
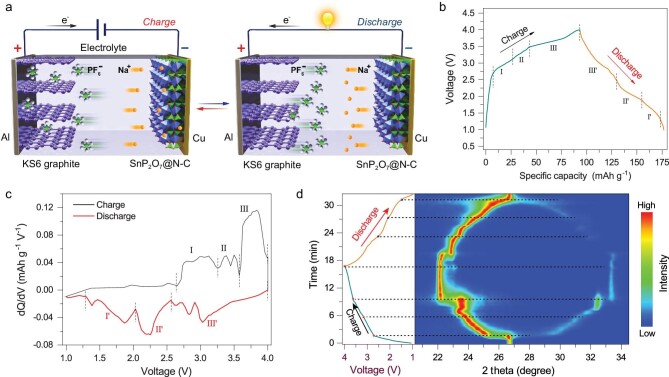
(a) Schematic illustration of the proof-of-concept Na-DIB configuration assembled with the SnP_2_O_7_@N-C anode and KS6 graphite cathode. (b) Galvanostatic charge–discharge profile of the Na-DIB in the voltage range of 1.0–4.0 V at 3 C, (c) corresponding dQ/dV differential curves, and (d) *in situ* XRD contour during charging/discharging process.

Consequently, we paired this anode with an environmentally friendly KS6 graphite cathode to construct a proof-of-concept Na-DIB to further explore its practical sodium storage capability in the full cell. Figure 5a schematically illustrates its working mechanism, where Na^+^ cations and PF_6_^−^ anions separately move to the SnP_2_O_7_@N-C anode and KS6 graphite cathode during the charging process, while both cations and anions return back to the electrolyte from the anode and cathode during discharging process, respectively. Its typical galvanostatic charge–discharge profile (Fig. 5b) in the voltage range of 1.0 to 4.0 V at 3 C (1 C = 100 mA g^−1^) exhibits several voltage plateaus, corresponding to the different intercalation/de-intercalation stages of PF_6_^–^ anions. According to the dQ/dV differential curve (Fig. 5c), the charging process (Fig. 5b) can be roughly separated into three voltage regions of 2.6–3.25 V (stage I), 3.25–3.55 V (stage II) and 3.55–4.0 V (stage III), corresponding to three different stages of anion intercalation into KS6 graphite cathode [50,52,53]. In order to get insight into the electrochemical process of the SnP_2_O_7_@N-C||KS6 Na-DIB during the charging process, the galvanostatic charge–discharge profile of Na||KS6 half-cell and corresponding dQ/dV differential curve (Supplementary Fig. S17) were provided. The dQ/dV differential curve also presents three different stages, indicating the dominant role of anion intercalation into KS6 graphite cathode. Conversely, a reverse evolution accompanies the discharging process, where different de-intercalation stages occur in voltage ranges of 4.0–2.6 V (stage III^′^), 2.6–2.06 V (stage II^′^) and 2.06–1.30 V (stage I^′^) in the discharging process (Fig. 5c and Supplementary Fig. S17). Such intercalation/de-intercalation behavior of PF_6_^–^ anions was further confirmed by the *in situ* XRD measurements during the charging/discharging process (Fig. 5d). The original XRD pattern presents a characteristic (002) peak of KS6 graphite cathode at 26.7°. In the charging process, the characteristic peak becomes weak and splits into two peaks individually shifting towards lower (main peak) and higher 2θ degrees, corresponding to the stage I of anion intercalation into KS6 graphite cathode. The stage II involves the formation of a stable intercalation phase at 23.6°. Then, the stage III relates to a sharp transition of diffraction peaks and the formation of another stable phase at 22.1°. Such peak evolution is ascribable to the successful intercalation of PF_6_^−^ anions into graphite cathode [79,80]. A reverse evolution occurs in the discharging process, and the two peaks gradually merge into the initial peak at 26.7° at the end of the discharging, indicating excellent reversibility of the intercalation/de-intercalation process of PF_6_^–^ anions into/from KS6 graphite cathode.

**Figure 6.**
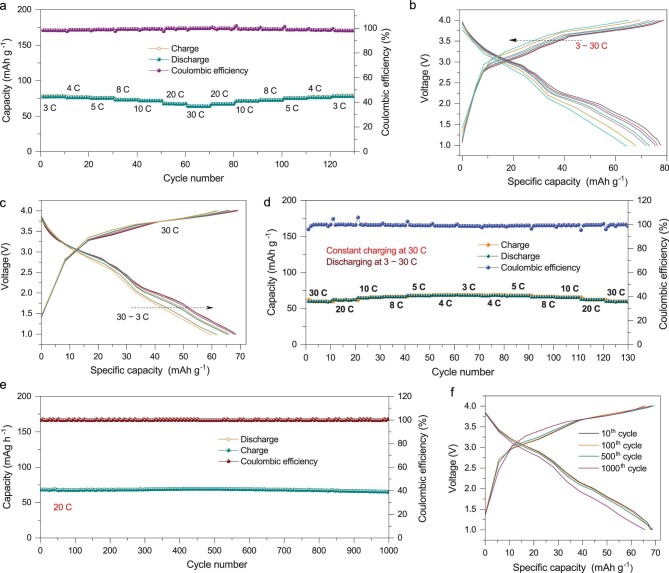
Electrochemical energy-storage performances of the proof-of-concept Na-DIB. (a) Rate capability and (b) corresponding galvanostatic charge–discharge profiles at different current densities. (c) Charge–discharge profiles at a constant charging current density of 30 C and different discharging rates and (d) the corresponding fast-charge/slow-discharge performance. (e) Long-term cycling stability and (f) the corresponding galvanostatic charge–discharge profiles at different cycles.

Figure 6a presents the rate capability of the SnP_2_O_7_@N-C||KS6 Na-DIB, which delivers a reversible discharge capacity of 78 mAh g^−1^ at 3 C. Even at 30 C, a specific capacity of 65 mAh g^−1^ can be obtained (83.3% capacity retention) with ∼100% Coulombic efficiency. The galvanostatic charge–discharge profiles at different current densities show similar shapes and a slight shift of voltage plateaus, indicating negligible electrochemical polarization (Fig. 6b). Besides, it can be rapidly charged at 30 C and slowly discharged down to 3 C (Fig. 6c and d). The discharge profiles exhibit a slight variation, and the corresponding specific capacity can be stably delivered even at different current densities, exhibiting a good fast-charge/slow-discharge ability. Moreover, the Na-DIB shows an excellent cycling performance with a capacity retention of ∼96% and a Coulombic efficiency of *∼*100% after 1000 cycles under a high rate of 20 C (Fig. 6e). The galvanostatic charge–discharge profiles at 10th, 100th, 500th and 1000th cycles have the same shape and voltage plateaus (Fig. 6f), further verifying its stable cycling ability. As shown in Supplementary Table S2, the SnP_2_O_7_@N-C||KS6 Na-DIB presents superior cycling performance, rate capability and Coulombic efficiency to previously reported Na-DIBs based on different anode materials [50,52–56,75,80–86].

## CONCLUSION

In summary, high-fraction (up to 95.6 wt%) SnP_2_O_7_ active anode material was successfully *in situ* implanted in the N-doped carbon matrix via a molecular grafting strategy. Such a synthesis strategy effectively enhanced the adhesion between active materials and carbon matrix, while the N doping led to high conductivity even at low C content. As a result, the anode showed a high specific capacity of ∼400 mAh g^−1^ at 0.1 A g^−1^, good rate performance up to 5.0 A g^−1^ and excellent cycling stability with a capacity retention of 92% after 1200 cycles at 1.5 A g^−1^. Furthermore, this anode was paired with an environmentally friendly KS6 graphite cathode to yield a proof-of-concept Na-DIB, showing a superior rate capability with a capacity retention of ∼83% even at a high current density of 30 C, good fast-charge/slow-discharge ability and long-term cycling life with a capacity retention of ∼96% after 1000 cycles at 20 C, exhibiting a great potential for high-performance Na-based energy storage devices.

## METHODS

### Synthesis of SnP_2_O_7_@N-C

SnCl_2_·2H_2_O powder was dissolved in deionized water under stirring, and then phytic acid solution and melamine powder were sequentially added into the above solution and subsequently stirred vigorously. Then the mixture was transferred to a two-necked flask and absolute ethanol was added and refluxed under stirring. Next, the obtained reaction product was collected by centrifugation, successively washed with deionized water and ethanol several times and dried under vacuum. Finally, the powder product was calcined in an Ar atmosphere to obtain a SnP_2_O_7_@N-C sample.

### Materials characterization

The morphological and elemental features were characterized using field-emission scanning electron microscope (FE-SEM). The FEI Tecnai G2 F30 was applied to acquire the transmission electron microscope (TEM) images, elemental mappings and SAED pattern. XRD analyses were implemented on a Rigaku D MiniFlex 600 diffractometer. Raman spectra were collected on Horiba LabRAM HR800. N_2_ physical adsorption-desorption analysis was carried out on ASAP 2020M. The chemical composition of SnP_2_O_7_/N-C sample was determined using XPS with monochromatic aluminum Kα radiation. TGA were conducted from 100°C to 700°C. FTIR of precursors and their composites were acquired using a PerkinElmer Frontier FTIR spectrophotometer. Tests about XANES were carried out at Synchrotron Light Research Institute (SLRI), Thailand.

### Electrochemical measurement

The electrochemical performance of the half-cells and DIB was carried out using CR2032 coin-type cells. The SnP_2_O_7_@N-C electrode was prepared by coating mixture slurry of the SnP_2_O_7_@N-C, Ketjenblack and carboxy methyl cellulose with a weight ratio of 70:20:10. For the half cells, the electrodes were pressed and punched into circular sheets 10 mm in diameter. The KS6 graphite cathode was prepared by mixing 80 wt% KS6 graphite, 10 wt% conductive carbon black and 10 wt% polyvinylidene fluoride (PVDF) to form a homogeneous slurry. In order to boost the full utilization of cathode material, the cathode sheet was punched into circular sheets 10 mm in diameter. The mass loading ratio of active anode/cathode materials for Na-DIB was ∼1 : 1 and the corresponding size of the anode sheet was 12 mm in diameter. Glass fabric was used as the separator, and 1 M NaClO_4_ in propylene carbonate (PC) with 5 wt% fluoroethylene carbonate (FEC) was used as the electrolyte for half cells. The electrolyte for the SnP_2_O_7_@N-C||KS6 DIB was 1 M NaPF_6_ dissolved in a mixture of ethylene carbonate (EC)/dimethyl carbonate (DMC)/ethyl methyl carbonate (EMC) (4 : 3 : 2 in volume). Cells were assembled in a glove box with water and oxygen content below 0.1 ppm and tested at room temperature. Galvanostatic charge–discharge tests and rate tests were conducted with a battery test system. EIS and CV were performed on an Autolab electrochemical workstation. All chemical reagents were used as received without any further purification. The capacity is calculated based on the mass of SnP_2_O_7_@N-C for half cells. The mass of KS6 is used to calculate the specific capacity of the DIB. More detailed materials are available in the online supplementary data.

## Supplementary Material

nwaa178_Supplemental_File

## References

[1] Zhang Y , Su Q, Xu W et al. A confined replacement synthesis of bismuth nanodots in MOF derived carbon arrays as binder-free anodes for sodium-ion batteries. Adv Sci 2019; 6: 1900162. 10.1002/advs.201900162 PMC6702623 31453056

[2] Li YQ , Lu YX, Meng QS et al. Regulating pore structure of hierarchical porous waste cork-derived hard carbon anode for enhanced Na storage performance. Adv Energy Mater 2019; 9: 1902852. 10.1002/aenm.201902852

[3] Jin T , Han Q, Jiao L. Binder-free electrodes for advanced sodium-ion batteries. Adv Mater 2020; 32: 1806304. 10.1002/adma.201806304 30811721

[4] Yao Y , Chen M, Xu R et al. CNT interwoven nitrogen and oxygen dual-doped porous carbon nanosheets as free-standing electrodes for high-performance Na-Se and K-Se flexible batteries. Adv Mater 2018; 30: 1805234. 10.1002/adma.201805234 30300459

[5] Huang Y , Wang Z, Jiang Y et al. Hierarchical porous Co_0.85_Se@reduced graphene oxide ultrathin nanosheets with vacancy-enhanced kinetics as superior anodes for sodium-ion batteries. Nano Energy 2018; 53: 524–35. 10.1016/j.nanoen.2018.09.010

[6] Xie D , Xia X, Zhong Y et al. Exploring advanced sandwiched arrays by vertical graphene and N-doped carbon for enhanced sodium storage. Adv Energy Mater 2017; 7: 1601804. 10.1002/aenm.201601804

[7] Song TY , Yao WJ, Kidkhunthod P et al. A low-cost and environmentally friendly mixed polyanionic cathode for sodium-ion storage. Angew Chem Int Ed 2020; 59: 740–5. 10.1002/anie.201912272 31591806

[8] Zhao RZ , Qian Z, Liu ZY et al. Molecular-level heterostructures assembled from layered black phosphorene and Ti_3_C_2_ MXene as superior anodes for high-performance sodium ion batteries. Nano Energy 2019; 65: 104037. 10.1016/j.nanoen.2019.104037

[9] Dong SH , Li CX, Li ZQ et al. Synergistic effect of porous phosphosulfide and antimony nanospheres anchored on 3D carbon foam for enhanced long-life sodium storage performance. Energy Storage Mater 2019; 20: 446–54. 10.1016/j.ensm.2018.10.024

[10] Kraychyk KV , Bhauriyal P, Piveteau L et al. High-energy-density dual-ion battery for stationary storage of electricity using concentrated potassium fluorosulfonylimide. Nat Commun 2018; 9: 4469. 10.1038/s41467-018-06923-6 30367050 PMC6203722

[11] Zhang M , Shoaib M, Fei H et al. Hierarchically porous N-doped carbon fibers as a free-standing anode for high-capacity potassium-based dual-ion battery. Adv Energy Mater 2019; 9: 1901663. 10.1002/aenm.201901663

[12] Ji B , Yao W, Zheng Y et al. A fluoroxalate cathode material for potassium-ion batteries with ultra-long cyclability. Nat Commun 2020; 11: 1225. 10.1038/s41467-020-15044-y 32144250 PMC7060185

[13] Zhao RZ , Di HX, Hui XB et al. Self-assembled Ti_3_C_2_ MXene and N-rich porous carbon hybrids as superior anodes for high-performance potassium-ion batteries. Energy Environ Sci 2020; 13: 246–57. 10.1039/C9EE03250A

[14] Li L , Liu LJ, Hu Z et al. Understanding high-rate K^+^-solvent co-intercalation in natural graphite for potassium-ion batteries. Angew Chem Int Ed 2020; 59: 12917–24. 10.1002/anie.202001966 32298024

[15] Liu QN , Hu Z, Liang YR et al. Facile synthesis of hierarchical hollow CoP@C composites with superior performance for sodium and potassium storage. Angew Chem Int Ed 2020; 59: 5159–64. 10.1002/anie.201913683 31849145

[16] Luo J , Xia Y, Zhang J et al. Enabling Mg metal anodes rechargeable in conventional electrolytes by fast ionic transport interphase. Natl Sci Rev 2020; 7: 333–41. 10.1093/nsr/nwz157 PMC8288991 34692049

[17] Zhou LM , Liu Q, Zhang ZH et al. Interlayer-spacing-regulated VOPO_4_ nanosheets with fast kinetics for high-capacity and durable rechargeable magnesium batteries. Adv Mater 2018; 30: 1801984. 10.1002/adma.201801984 29939435

[18] Wang W , Liu L, Wang PF et al. A novel bismuth-based anode material with a stable alloying process by the space confinement of an in situ conversion reaction for a rechargeable magnesium ion battery. Chem Commun 2018; 54: 1714–7. 10.1039/C7CC08206A 29243767

[19] Wang M , Jiang C, Zhang S et al. Reversible calcium alloying enables a practical room-temperature rechargeable calcium-ion battery with a high discharge voltage. Nat Chem 2018; 10: 667–72. 10.1038/s41557-018-0045-4 29686378

[20] Wu N , Yao W, Song X et al. A calcium-ion hybrid energy storage device with high capacity and long cycling life under room temperature. Adv Energy Mater 2019; 9: 1803865. 10.1002/aenm.201803865

[21] Yang W , Dong L, Yang W et al. 3D oxygen-defective potassium vanadate/carbon nanoribbon networks as high-performance cathodes for aqueous zinc-ion batteries. Small Methods 2020; 4: 1900670. 10.1002/smtd.201900670

[22] Wu Z-S , Bao X, Sun C et al. Scalable fabrication of printed Zn//MnO_2_ planar micro-batteries with high volumetric energy density and exceptional safety. Natl Sci Rev 2020; 7: 64–72. 10.1093/nsr/nwz070 PMC8288951 34692018

[23] Hao J , Long J, Li B et al. Toward high-performance hybrid Zn-based batteries via deeply understanding their mechanism and using electrolyte additive. Adv Funct Mater 2019; 29: 1903605. 10.1002/adfm.201903605

[24] Wang H , Wang M, Tang Y. A novel zinc-ion hybrid supercapacitor for long-life and low-cost energy storage applications. Energy Storage Mater 2018; 13: 1–7. 10.1016/j.ensm.2017.12.022

[25] Li N , Li GQ, Li CJ et al. Bi-cation electrolyte for a 1.7 V aqueous Zn ion battery. ACS Appl Mater Interfaces 2020; 12: 13790–6. 10.1021/acsami.9b20531 32108465

[26] Zhang Y , Liu SQ, Ji YJ et al. Emerging nonaqueous aluminum-ion batteries: challenges, status, and perspectives. Adv Mater 2018; 30: 1706310. 10.1002/adma.201706310 29920792

[27] Yang HC , Li HC, Li J et al. The rechargeable aluminum battery: opportunities and challenges. Angew Chem Int Ed 2019; 58: 11978–96. 10.1002/anie.201814031 30687993

[28] Zhang X , Zhang G, Wang S et al. Porous CuO microsphere architectures as high-performance cathode materials for aluminum-ion batteries. J Mater Chem A 2018; 6: 3084–90. 10.1039/C7TA10632G

[29] Niu YB , Yin YX, Guo YG. Nonaqueous sodium-ion full cells: status, strategies, and prospects. Small 2019; 15: 1900233. 10.1002/smll.201900233 30908817

[30] Hou HS , Qiu XQ, Wei WF et al. Carbon anode materials for advanced sodium-ion batteries. Adv Energy Mater 2017; 7: 1602898. 10.1002/aenm.201602898

[31] Zhao C , Liu L, Qi X et al. Solid-state sodium batteries. Adv Energy Mater 2018; 8: 1703012. 10.1002/aenm.201703012

[32] Lim E , Jo C, Kim MS et al. High-performance sodium-ion hybrid supercapacitor based on Nb_2_O_5_@carbon core–shell nanoparticles and reduced graphene oxide nanocomposites. Adv Funct Mater 2016; 26: 3711–9. 10.1002/adfm.201505548

[33] Huang Y , Zhao L, Li L et al. Electrolytes and electrolyte/electrode interfaces in sodium-ion batteries: from scientific research to practical application. Adv Mater 2019; 31: 1808393. 10.1002/adma.201808393 30920698

[34] Chen M , Xiao J, Hua W et al. Strategy of cation and anion dual doping for potential elevating of titanium redox for high-power sodium-ion batteries. Angew Chem Int Ed 2020; 59: 12076–83. 10.1002/anie.202003275 32249496

[35] Jiang C , Fang Y, Zhang W et al. A multi-ion strategy towards rechargeable sodium-ion full batteries with high working voltage and rate capability. Angew Chem Int Ed 2018; 57: 16370–4. 10.1002/anie.201810575 30320428

[36] Wang WL , Gang Y, Hu Z et al. Reversible structural evolution of sodium-rich rhombohedral Prussian blue for sodium-ion batteries. Nat Commun 2020; 11: 980 10.1038/s41467-020-14444-4 32080172 PMC7033191

[37] Xu X , Lin K, Zhou D et al. Quasi-solid-state dual-ion sodium metal batteries for low-cost energy storage. Chem 2020; 6: 902–18. 10.1016/j.chempr.2020.01.008

[38] Sui YM , Liu CF, Masse RC et al. Dual-ion batteries: the emerging alternative rechargeable batteries. Energy Storage Mater 2020; 25: 1–32. 10.1016/j.ensm.2019.11.003

[39] Zhou X , Liu Q, Jiang C et al. Strategies towards low-cost dual-ion batteries with high performance. Angew Chem Int Ed 2020; 59: 3802–32. 10.1002/anie.201814294 30865353

[40] Kravchyk KV , Kovalenko MV. Rechargeable dual-ion batteries with graphite as a cathode: key challenges and opportunities. Adv Energy Mater 2019; 9: 1901749. 10.1002/aenm.201901749

[41] Hao J , Li X, Song X et al. Recent progress and perspectives on dual-ion batteries. EnergyChem 2019; 1: 100004. 10.1016/j.enchem.2019.100004

[42] Han X , Xu G, Zhang Z et al. An in situ interface reinforcement strategy achieving long cycle performance of dual-ion batteries. Adv Energy Mater 2019; 9: 1804022. 10.1002/aenm.201804022

[43] Ji B , Zhang F, Wu N et al. A dual-carbon battery based on potassium-ion electrolyte. Adv Energy Mater 2017; 7: 1700920. 10.1002/aenm.201700920

[44] Wu S , Zhang F, Tang Y. A novel calcium-ion battery based on dual-carbon configuration with high working voltage and long cycling life. Adv Sci 2018; 5: 1701082. 10.1002/advs.201701082 PMC6097003 30128228

[45] Fan L , Liu Q, Chen S et al. Potassium-based dual ion battery with dual-graphite electrode. Small 2017; 13: 1701011. 10.1002/smll.201701011 28597529

[46] Zhu JJ , Li YL, Yang BJ et al. A dual carbon-based potassium dual ion battery with robust comprehensive performance. Small 2018; 14: 1801836. 10.1002/smll.201801836 29971944

[47] Rodríguez-Pérez IA , Ji X. Anion hosting cathodes in dual-ion batteries. ACS Energy Lett 2017; 2: 1762–70. 10.1021/acsenergylett.7b00321

[48] Liu M , Xing L, Xu K et al. Deciphering the paradox between the co-intercalation of sodium-solvent into graphite and its irreversible capacity. Energy Storage Mater 2020; 26: 32–9. 10.1016/j.ensm.2019.12.026

[49] Xu ZL , Yoon G, Park KY et al. Tailoring sodium intercalation in graphite for high energy and power sodium ion batteries. Nat Commun 2019; 10: 2598. 10.1038/s41467-019-10551-z 31197187 PMC6565630

[50] Sheng M , Zhang F, Ji B et al. A novel tin-graphite dual-ion battery based on sodium-ion electrolyte with high energy density. Adv Energy Mater 2017; 7: 1601963. 10.1002/aenm.201601963

[51] Xie D , Zhang M, Wu Y et al. A flexible dual-ion battery based on sodium-ion quasi-solid-state electrolyte with long cycling life. Adv Funct Mater 2020; 30: 1906770. 10.1002/adfm.201906770

[52] Zhu H , Zhang F, Li J et al. Penne-like MoS_2_/carbon nanocomposite as anode for sodium-ion-based dual-ion battery. Small 2018; 14: 1703951. 10.1002/smll.201703951 29399964

[53] Li ZY , Yang LW, Xu GB et al. Hierarchical MoS_2_@N-doped carbon hollow spheres with enhanced performance in sodium dual-ion batteries. ChemElectroChem 2019; 6: 661–7. 10.1002/celc.201801282

[54] Liu Y , Hu X, Zhong G et al. Layer-by-layer stacked nanohybrids of N,S-co-doped carbon film modified atomic MoS_2_ nanosheets for advanced sodium dual-ion batteries. J Mater Chem A 2019; 7: 24271–80. 10.1039/C9TA09636A

[55] Wang X , Qi L, Wang H. Anatase TiO_2_ as a Na^+^-storage anode active material for dual-ion batteries. ACS Appl Mater Interfaces 2019; 11: 30453–9. 10.1021/acsami.9b09703 31355628

[56] Li C , Wang X, Li J et al. FePO_4_ as an anode material to obtain high-performance sodium-based dual-ion batteries. Chem Commun 2018; 54: 4349–52. 10.1039/C7CC09714J 29645023

[57] Zhao D , Zhao R, Dong S et al. Alkali-induced 3D crinkled porous Ti_3_C_2_ MXene architectures coupled with NiCoP bimetallic phosphide nanoparticles as anodes for high-performance sodium-ion batteries. Energy Environ Sci 2019; 12: 2422–32. 10.1039/C9EE00308H

[58] Li W , Yao Z, Zhong Y et al. Enhancement of the advanced Na storage performance of Na_3_V_2_(PO_4_)_3_ in a symmetric sodium full cell via a dual strategy design. J Mater Chem A 2019; 7: 10231–8. 10.1039/C9TA02041A

[59] Ge P , Li S, Xu L et al. Hierarchical hollow-microsphere metal–selenide@carbon composites with rational surface engineering for advanced sodium storage. Adv Energy Mater 2019; 9: 1803035. 10.1002/aenm.201803035

[60] Zhang SW , Lv W, Qiu D et al. An ion-conducting SnS-SnS_2_ hybrid coating for commercial activated carbons enabling their use as high performance anodes for sodium-ion batteries. J Mater Chem A 2019; 7: 10761–8. 10.1039/C9TA00599D

[61] Wang MY , Wang XL, Yao ZJ et al. SnO_2_ nanoflake arrays coated with polypyrrole on a carbon cloth as flexible anodes for sodium-ion batteries. ACS Appl Mater Interfaces 2019; 11: 24198–204. 10.1021/acsami.9b08378 31199106

[62] Wang Y , Zhang Y, Shi J et al. Tin sulfide nanoparticles embedded in sulfur and nitrogen dual-doped mesoporous carbon fibers as high-performance anodes with battery-capacitive sodium storage. Energy Storage Mater 2019; 18: 366–74. 10.1016/j.ensm.2018.08.014

[63] Zhao J , Wang G, Hu R et al. Ultrasmall-sized SnS nanosheets vertically aligned on carbon microtubes for sodium-ion capacitors with high energy density. J Mater Chem A 2019; 7: 4047–54. 10.1039/C9TA00141G

[64] Xia J , Liu L, Jamil S et al. Free-standing SnS/C nanofiber anodes for ultralong cycle-life lithium-ion batteries and sodium-ion batteries. Energy Storage Mater 2019; 17: 1–11. 10.1016/j.ensm.2018.08.005

[65] Li ZQ , Ge XL, Li CX et al. Rational microstructure design on metal–organic framework composites for better electrochemical performances: design principle, synthetic strategy, and promotion mechanism. Small Methods 2020; 4: 1900756. 10.1002/smtd.201900756

[66] Pan J , Chen S, Zhang D et al. SnP_2_O_7_ covered carbon nanosheets as a long-life and high-rate anode material for sodium-ion batteries. Adv Funct Mater 2018; 28: 1804672. 10.1002/adfm.201804672

[67] Wang ZZ , Lv P, Hu Y et al. Thermal degradation study of intumescent flame retardants by TG and FTIR: melamine phosphate and its mixture with pentaerythritol. J Anal Appl Pyrolysis 2009; 86: 207–14. 10.1016/j.jaap.2009.06.007

[68] Li Q , Li Z, Zhang Z et al. Low-temperature solution-based phosphorization reaction route to Sn_4_P_3_/reduced graphene oxide nanohybrids as anodes for sodium ion batteries. Adv Energy Mater 2016; 6: 1600376. 10.1002/aenm.201600376

[69] Sha M , Zhang H, Nie YT et al. Sn nanoparticles@nitrogen-doped carbon nanofiber composites as high-performance anodes for sodium-ion batteries. J Mater Chem A 2017; 5: 6277–83. 10.1039/C7TA00690J

[70] Yang X , Zhang R-Y, Zhao J et al. Amorphous tin-based composite oxide: a high-rate and ultralong-life sodium-ion-storage material. Adv Energy Mater 2018; 8: 1701827. 10.1002/aenm.201701827

[71] Liu Y , Zhang N, Jiao L et al. Tin nanodots encapsulated in porous nitrogen-doped carbon nanofibers as a free-standing anode for advanced sodium-ion batteries. Adv Mater 2015; 27: 6702–7. 10.1002/adma.201503015 26422696

[72] Guo WY , Ding K, Mei SX et al. Hollow spheres consisting of SnS nanosheets conformally coated with S-doped carbon for advanced lithium-/sodium-ion battery anodes. ChemElectroChem 2020; 7: 914–21. 10.1002/celc.201901923

[73] Liu Z , Handa K, Kaibuchi K et al. Comparison of the Sn L edge X-ray absorption spectra and the corresponding electronic structure in Sn, SnO, and SnO_2_. J Electron Spectrosc 2004; 135: 155–8. 10.1016/j.elspec.2004.03.002

[74] Pelliccione CJ , Timofeeva EV, Segre CU. Potential-resolved in situ X-ray absorption spectroscopy study of Sn and SnO_2_ nanomaterial anodes for lithium-ion batteries. J Phys Chem C 2016; 120: 5331–9. 10.1021/acs.jpcc.5b12279

[75] Dong S , Li Z, Rodríguez-Pérez IA et al. A novel coronene//Na_2_Ti_3_O_7_ dual-ion battery. Nano Energy 2017; 40: 233–9. 10.1016/j.nanoen.2017.08.022

[76] Li BQ , Liu Y, Jin X et al. Designed formation of hybrid nanobox composed of carbon sheathed CoSe_2_ anchored on nitrogen-doped carbon skeleton as ultrastable anode for sodium-ion batteries. Small 2019; 15: 1902881. 10.1002/smll.201902881 31433124

[77] Bai YL , Xarapatgvl R, Wu XY et al. Core-shell anatase anode materials for sodium-ion batteries: the impact of oxygen vacancies and nitrogen-doped carbon coating. Nanoscale 2019; 11: 17860–8. 10.1039/C9NR06245A 31553002

[78] Ying HJ , Zhang SL, Meng Z et al. Ultrasmall Sn nanodots embedded inside N-doped carbon microcages as high-performance lithium and sodium ion battery anodes. J Mater Chem A 2017; 5: 8334–42. 10.1039/C7TA01480E

[79] Li N , Zhang F, Tang YB. Hierarchical T-Nb_2_O_5_ nanostructure with hybrid mechanisms of intercalation and pseudocapacitance for potassium storage and high-performance potassium dual-ion batteries. J Mater Chem A 2018; 6: 17889–95. 10.1039/C8TA07987K

[80] Hu Z , Liu Q, Zhang K et al. All carbon dual ion batteries. ACS Appl Mater Interfaces 2018; 10: 35978–83. 10.1021/acsami.8b11824 30207686

[81] Yao X , Ke Y, Ren W et al. Defect-rich soft carbon porous nanosheets for fast and high-capacity sodium-ion storage. Adv Energy Mater 2019; 9: 1803260. 10.1002/aenm.201803260

[82] Ma R , Fan L, Chen S et al. Offset initial sodium loss to improve coulombic efficiency and stability of sodium dual-ion batteries. ACS Appl Mater Interfaces 2018; 10: 15751–9. 10.1021/acsami.8b03648 29664614

[83] Wang X , Zheng C, Qi L et al. Carbon derived from pine needles as a Na^+^-storage electrode material in dual-ion batteries. Glob Chall 2017; 1: 1700055. 10.1002/gch2.201700055 31565289 PMC6607337

[84] Fan L , Liu Q, Chen S et al. Soft carbon as anode for high-performance sodium-based dual ion full battery. Adv Energy Mater 2017; 7: 1602778. 10.1002/aenm.201602778

[85] Aubrey ML , Long JR. A dual-ion battery cathode via oxidative insertion of anions in a metal-organic framework. J Am Chem Soc 2015; 137: 13594–602. 10.1021/jacs.5b08022 26436465

[86] Fan J , Fang Y, Xiao Q et al. A dual-ion battery with a ferric ferricyanide anode enabling reversible Na^+^ intercalation. Energy Technol 2019; 7: 1800978. 10.1002/ente.201800978

