# Supplementary material for: Molecular grafting towards high-fraction active nanodots implanted in N-doped carbon for sodium dual-ion batteries
Source: Natl Sci Rev. 2020 Aug 25;8(7):nwaa178. doi: 10.1093/nsr/nwaa178 (PMC8310755; doi:10.1093/nsr/nwaa178)
Supplement: nwaa178_Supplemental_File [file nwaa178_supplemental_file.docx]

**Supplementary Information for**

**Molecular Grafting towards High-Fraction Active Nanodots Implanted in N-doped Carbon for Sodium Dual-Ion Batteries**

Sainan Mu^a,b†^, Qirong Liu^a†^, Pinit Kidkhunthod^c^, Xiaolong Zhou^a^, Wenlou Wang^b^, Yongbing Tang^a^*

^a^Functional Thin Films Research Center, Shenzhen Institutes of Advanced Technology, Chinese Academy of Sciences, Shenzhen, 518055, China

^b^Nano Science and Technology Institute, University of Science and Technology of China, Suzhou, 215123, China

^c^Synchrotron Light Research Institute, 111 University Avenue, Muang District, Nakhon Ratchasima 30000, Thailand

^†^S.N. Mu and Q.R. Liu equally contributed to this work.

*Corresponding authors: E-mails: tangyb@siat.ac.cn

**METHODS**

**Synthesis of SnP_2_O_7_**@**N-C**

In a typical synthesis, 10 mmol SnCl_2_·2H_2_O powder (99%, Aladdin Ltd.) was dissolved in 50 ml deionized water under stirring, and then 2 mmol phytic acid solution (70%, Aladdin Ltd.) and 8 mmol melamine powder (99%, Aladdin Ltd.) were sequentially added into the above solution and subsequently stirred vigorously at 55 °C for 2 h. Then, the mixture was transferred to a 250 mL two-necked flask, and 50 mL absolute ethanol was added, and refluxed at 85 °C for 12 h under stirring. Next, after cooling to room temperature, the obtained reaction product was collected by centrifugation, successively washed with deionized water and ethanol several times and dried at 80°C for 12 h under vacuum. Finally, the powder product was calcined at 600°C for 2 h in an Ar atmosphere to obtain SnP_2_O_7_@N-C sample. For comparison, the SnP_2_O_7_@C sample without N doping was also synthesized by the same synthetic procedure without adding melamine. And the pure SnP_2_O_7_ sample was prepared by the calcination treatment of the SnP_2_O_7_@N-C at 600 ^o^C for 1 h under an ambient atmosphere. All chemical reagents were used as received without any further purification.

**Materials characterization**

The morphological and elemental features were characterized using field-emission scanning electron microscope (FE-SEM, HITACHI S4800). The FEI Tecnai G2 F30 was applied to acquire the transmission electron microscope (TEM) images, elemental mappings and selected area electron diffraction (SAED) pattern. X-ray diffraction (XRD) analyses were implemented on a Rigaku D MiniFlex 600 diffractometer with Cu Kα radiation (20 kV, λ = 0.154056 nm) at a scanning rate of 8° min^–1^. Raman spectra were collected with 532 nm excitation on Horiba LabRAM HR800. N_2_ physical adsorption-desorption analysis was carried out on ASAP 2020M. The chemical composition of SnP_2_O_7_/N-C sample was determined using X-ray photoelectron spectroscopy (XPS, ESCALAB 250Xi, Thermo Fisher) with monochromatic aluminum Kα radiation. Electrodes for TEM test were washed with DMC and sealed in an Ar-filled glove box. Thermogravimetric analysis (TGA, Q600 SDT) were conducted from 100 ^o^C to 700 °C at a heating rate of 5 °C min^-1^ in air. Prior to the TGA test, the sample was dehydrated at 150 ^o^C for 10 min. Fourier transform infrared spectra (FTIR) of precursors and their composites were acquired using on a PerkinElmer Frontier FTIR spectrophotometer. Tests about X-ray absorption near-edge spectra (XANES) were carried out at Synchrotron Light Research Institute (SLRI, public organization), Thailand. The beamline photon source covers an energy range of 40-1040 eV at the resolving power of 10,000. The synchrotron radiation source at the storage ring was generated using a beam energy of 1.2 GeV. Sn L_3_-edge XANES spectra of the samples were collected at the SUTNANOTEC-SLRI XAS beamline (BL5.2). The typical loading of SnP_2_O_7_/N-C anode was 6~7 mg cm^−2^.

**Electrochemical measurement**

The electrochemical performance of the half-cells and dual-ion battery (DIB) were carried out using CR2032 coin-type cells. The SnP_2_O_7_@N-C electrode was prepared by coating mixture slurry of the SnP_2_O_7_@N-C, Ketjenblack (ECP-600JD, Lion Corporation) and carboxy methyl cellulose (Aladdin Ltd.) with a weight ratio of 70:20:10. The mixture were firstly ground with some H_2_O solvent together to form uniform slurry and then coated onto copper foil and dried at 80 °C in vacuum for 12 h. For the half cells, the electrodes were pressed and punched into circular sheets with 10 mm in diameter with mass loading (active material) of ~1.5 mg cm^-2^.The KS6 graphite (Timcal) cathode was prepared by mixing 80 wt% KS6 graphite, 10 wt% conductive carbon black (Shenzhen Kejing Star Technology Ltd.), 10 wt% polyvinylidene fluoride (PVDF, Shenzhen Kejing Star Technology Ltd.) and several drops of N-methyl-2-pyrrolidone (NMP, Shenzhen Kejing Star Technology Ltd.) solvent together to form a homogeneous slurry. The slurry was coated onto Al foil and dried at 80 ^o^C in vacuum for overnight. In order to boost the full utilization of cathode material, the cathode sheet was then pressed and punched into circular sheets with 10 mm in diameter with a mass loading of ~1.2 mg cm^-2^. The mass loading ratio of active anode/cathode materials for Na-DIB was ~1:1 and the corresponding size of anode sheet was 12 mm in diameter. Glass fabric (Whatman, GF/A, 47 mm in diameter, Shanghai Huanao Technology Ltd.) was used as the separator, and 1 M NaClO_4_ (99.99%, Aladdin Ltd.) in propylene carbonate (PC, 99.95%) with 5 wt% fluoroethylene carbonate (FEC, 99.95%, Dodochem) was used as the electrolyte for half cells. The electrolyte for the SnP_2_O_7_@N-C||KS6 DIB was 1 M NaPF_6_ (99.98%, Dodochem) dissolved in a mixture of ethylene carbonate (EC, 99.95%, Dodochem)/dimethyl carbonate (DMC, 99.95%, Dodochem)/ethyl methyl carbonate (EMC, 99.95%, Dodochem) (4:3:2 in volume). Cells were assembled in a glove box (MIKROUNA universal 2440-750) with water and oxygen content below 0.1 ppm and tested at room temperature. Galvanostatic charge-discharge tests and rate tests were conducted with a battery test system (NEWARE CT-4008). Electrochemical impedance spectroscopy (EIS) and cyclic voltammetry (CV) were performed on an Autolab (PGSTAT302N, Switzerland) electrochemical workstation. All chemical reagents were used as received without any further purification. The capacity is calculated based on the mass of SnP_2_O_7_@N-C for half cells. The mass of KS6 is used to calculate the specific capacity of the DIB.


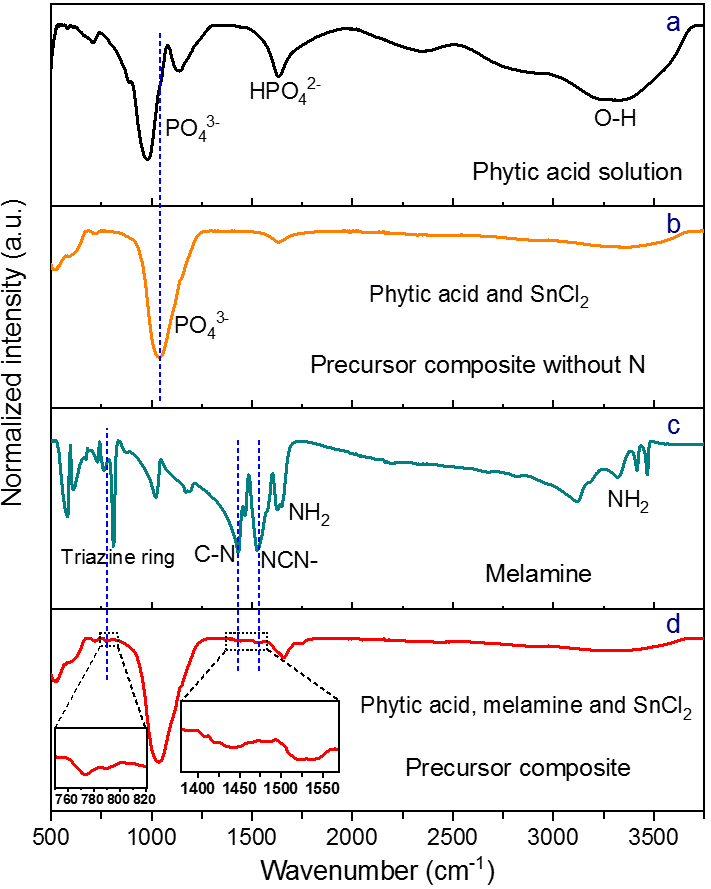


**Figure S1. FTIR spectra of phytic acid solution (a), precursor composite without the addition of melamine (b), melamine (c), and precursor composite (d).**


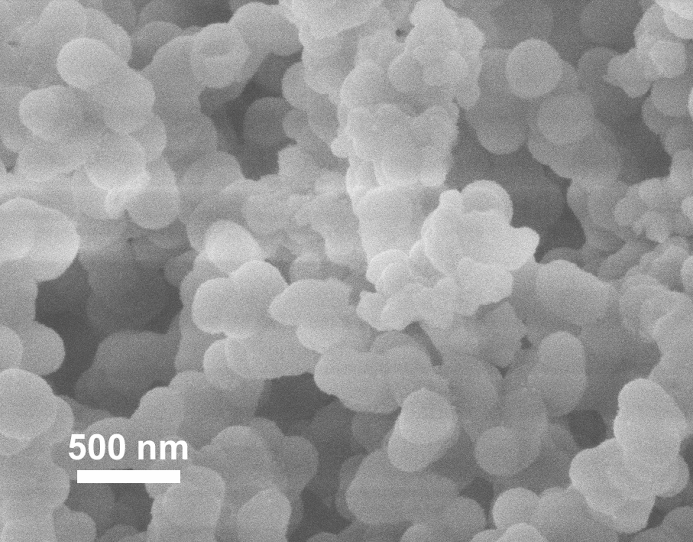


**Figure S2. Typical SEM image of molecular grafting precursor composite.**


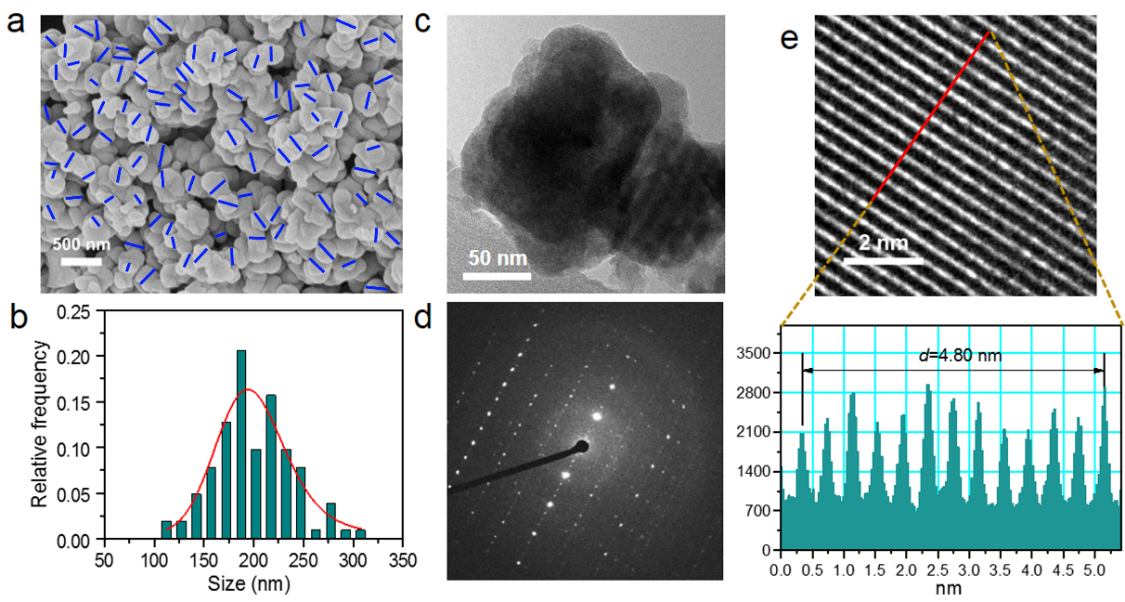


**Figure S3. (a-b) Statistic size distribution of SnP_2_O_7_@N-C nanoparticles, (c) TEM image and (d) corresponding SAED pattern, (e) HRTEM denoting lattice fringe distance (*d* = 0.40 nm) acquired along the red line.**


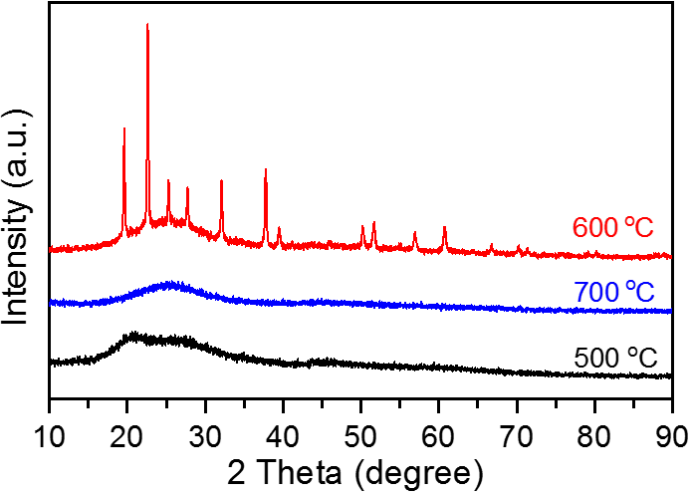


**Figure S4. XRD patterns of samples synthesized at different calcination temperatures.**


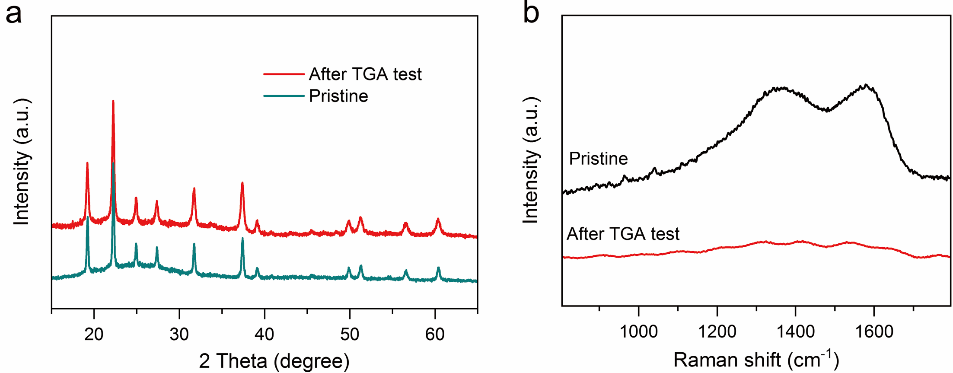


**Figure S5. (a) XRD patterns and (b) Raman spectra of SnP_2_O_7_@N-C sample before and after TGA test.**


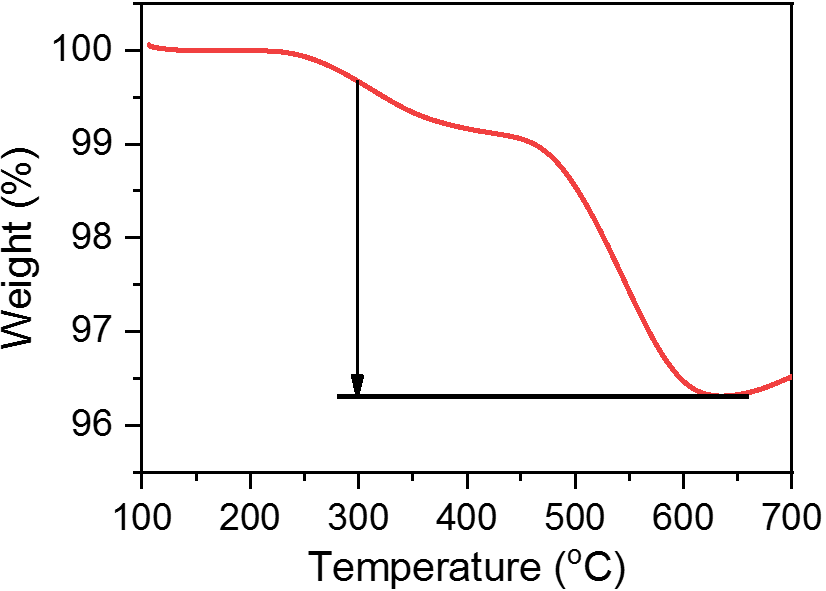


**Figure S6. TGA analysis of as-synthesized SnP_2_O_7_@C.**


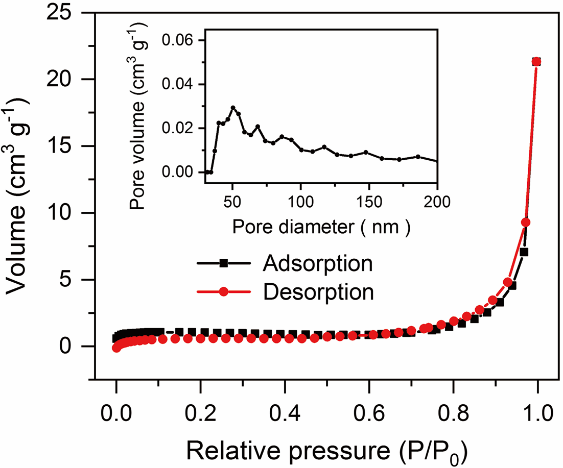


**Figure S7. Nitrogen adsorption–desorption isotherm profile and the corresponding pore size distribution (inset) of SnP_2_O_7_@N-C.**


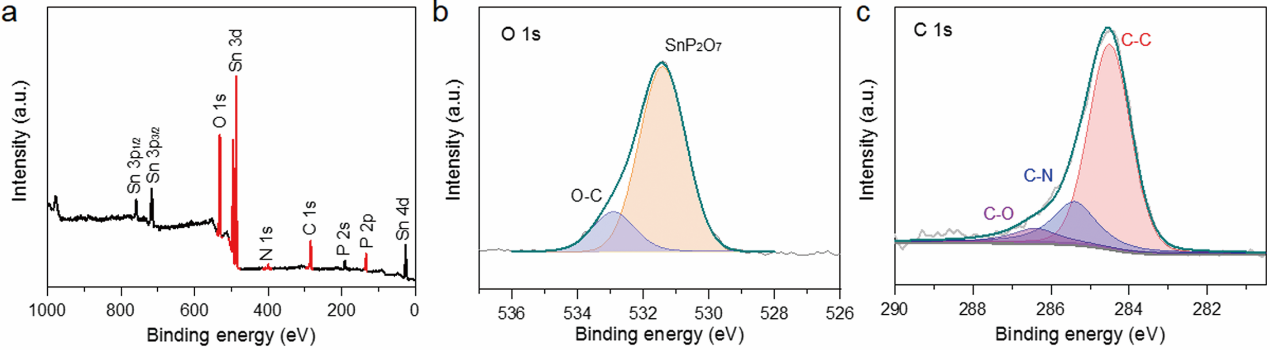


**Figure S8. (a) XPS survey spectrum, and high-resolution O 1s (b) and C 1s (c) XPS spectra.**


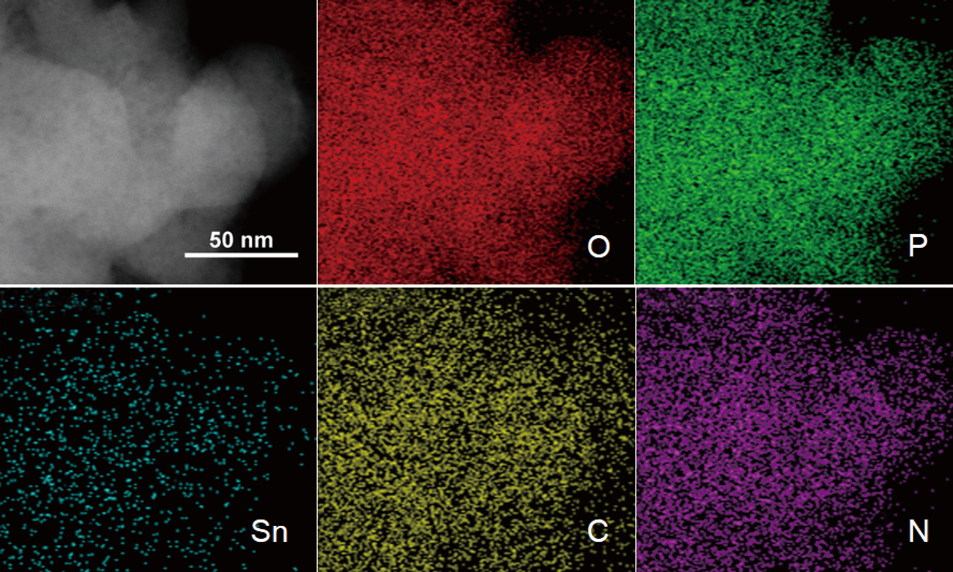


**Figure S9. Energy dispersive X-ray spectroscopy (EDX) mappings of SnP_2_O_7_@N-C composite.**


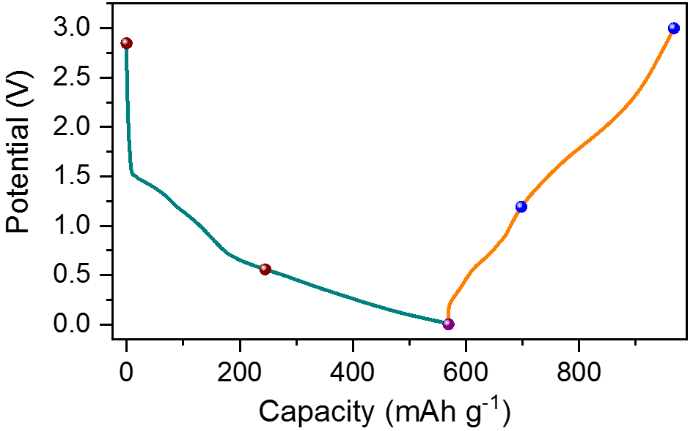


**Figure S10. Positions at the first galvanostatic charge-discharge profile chose for XANES and HRTEM characterizations of SnP_2_O_7_@N-C.**


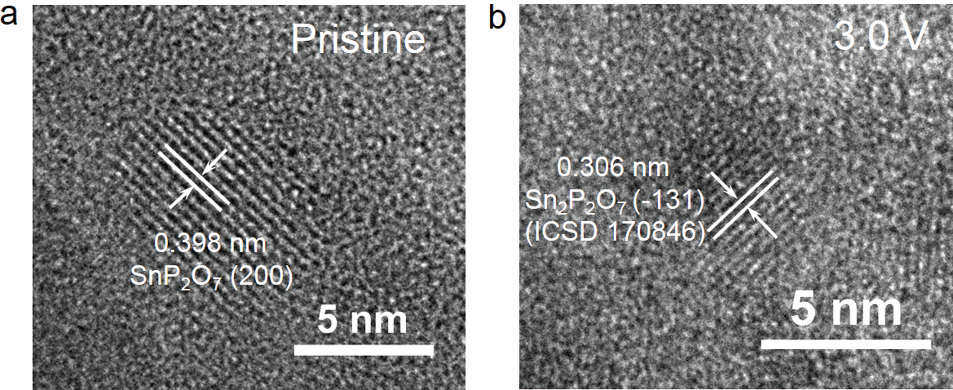


**Figure S11. HRTEM images of pristine and fully desodiated SnP_2_O_7_@N-C.**


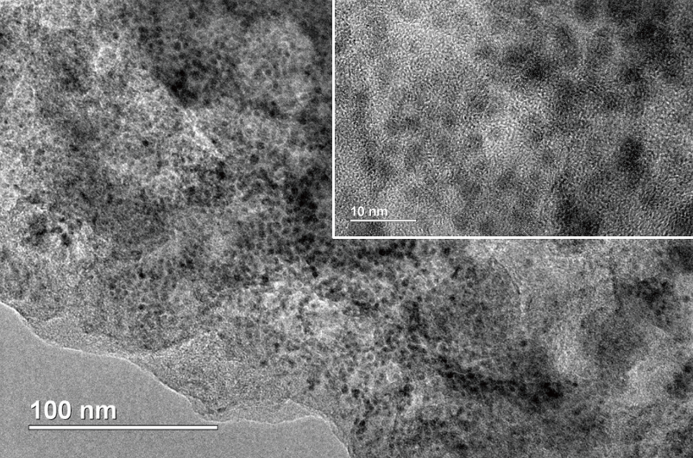


**Figure S12. TEM images of fully sodiated SnP_2_O_7_@N-C after the first cycle.**


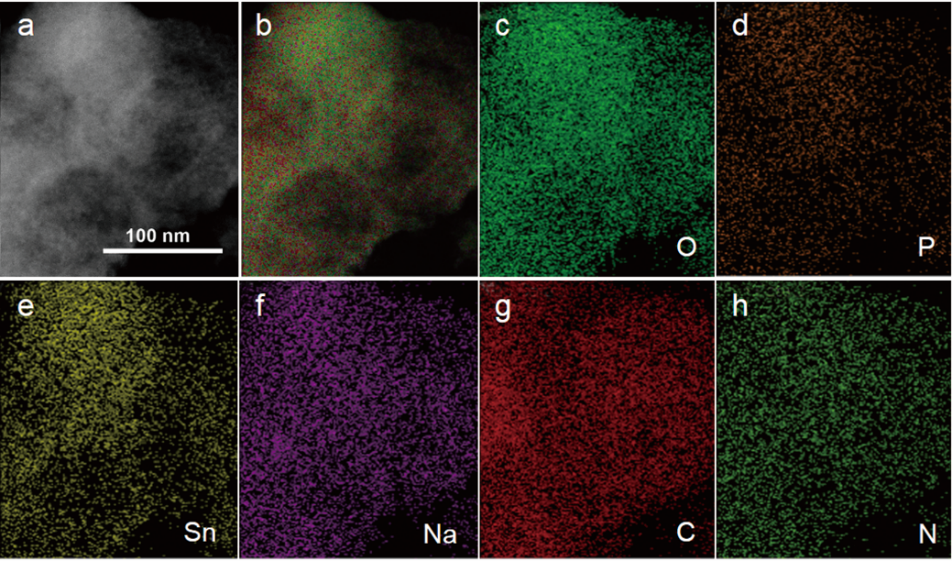


**Figure S13. EDX mappings of the fully sodiated SnP_2_O_7_@N-C:** (a) selected area, (b) overlapped EDX mapping of all elements, (c-h) individual EDX mappings of O, P, Sn, Na, C and N elements.


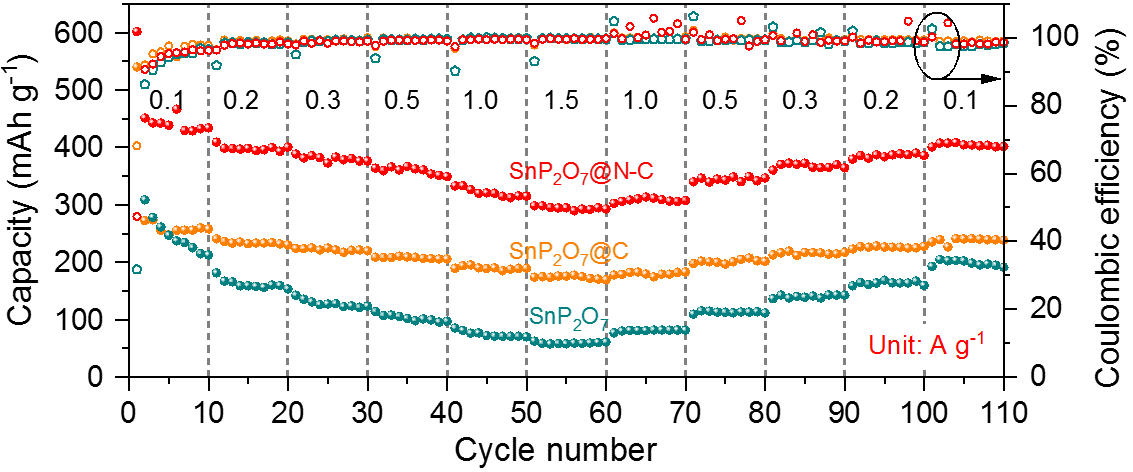


**Figure S14. Comparison among rate capabilities of SnP_2_O_7_@N-C, SnP_2_O_7_@C and pure SnP_2_O_7_ in the potential range of 0.01-3.0 V.**


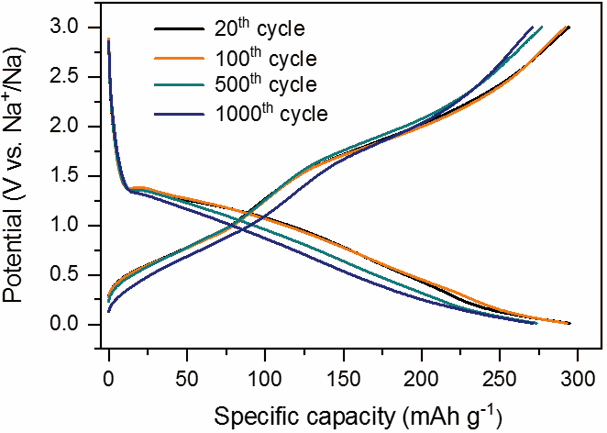


**Figure S15. Galvanostatic charge-discharge profiles of SnP_2_O_7_@N-C at different cycles (1.5 A g^−1^).**


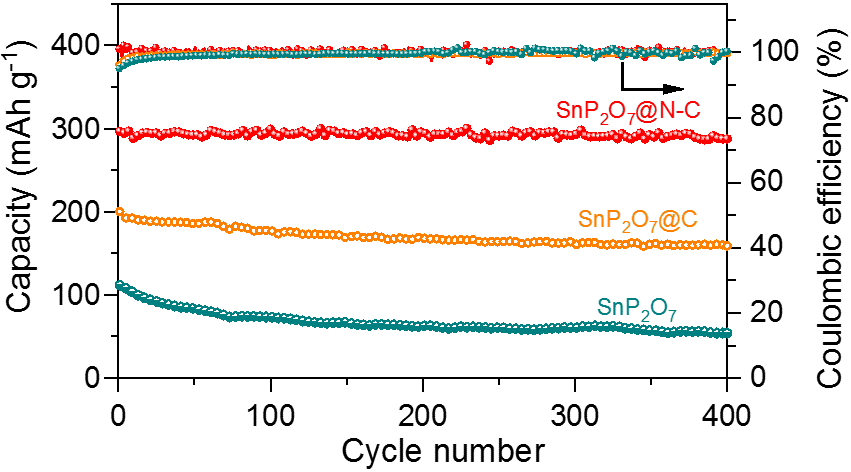


**Figure S16. Comparison of cycling performances of SnP_2_O_7_@N-C, SnP_2_O_7_@C and SnP_2_O_7_** **at 1.5 A g^-1^.**


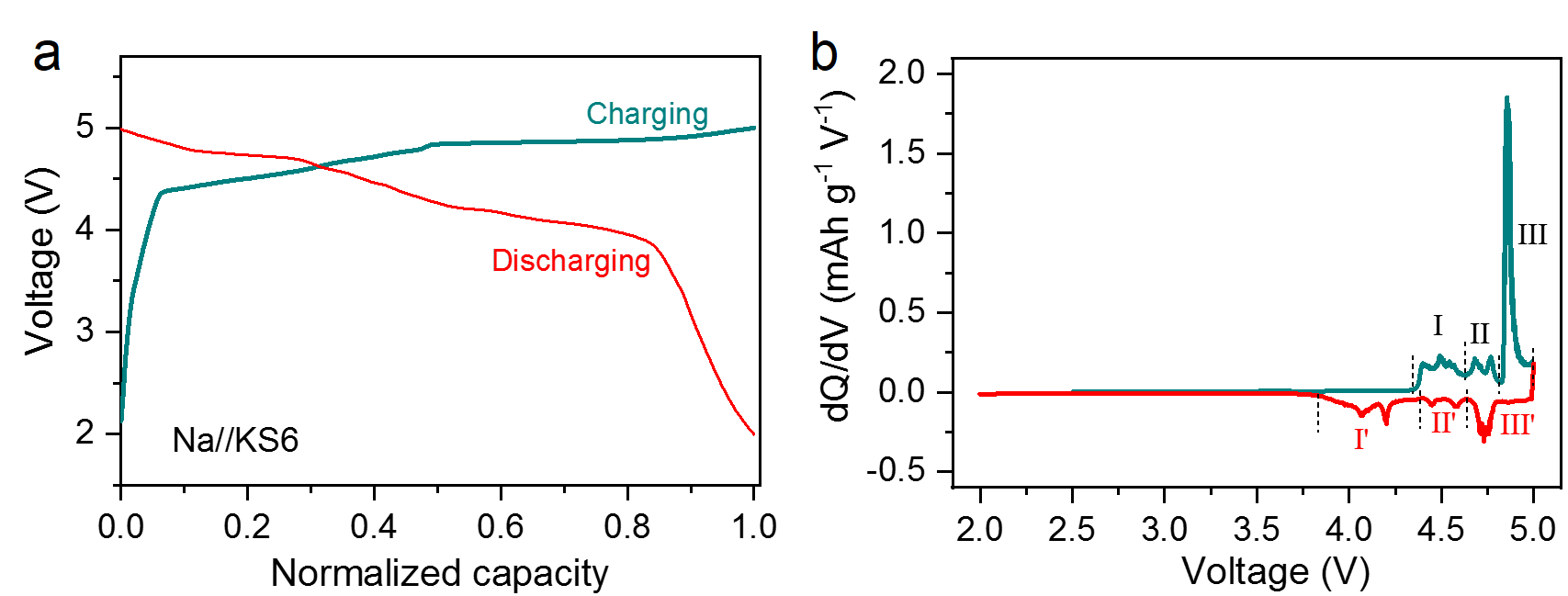


Figure S17. (a) Typical galvanostatic charge-discharge profile and (b) corresponding dQ/dV differential curve of the Na//KS6 half-cell.

**Table S1. Comparison of carbon content and electrochemical performances of Sn-based compound/carbon composite anodes for SIBs.**

| Composite anode | Carbon content (wt%) | Specific capacity / current density  (mAh g^-1^/A g^−1^) | Cycling performance  (cycles/capacity retention) |
| --- | --- | --- | --- |
| SnO_2-x_/C [1] | 46 % | 634/0.1 | 2000/100% |
| C@SnS/SnO_2_@Gr [2] | 24 % | 713/0.03 | 500/73% |
| SnS_2_ NC/EDA–RGO [3] | 25 % | 680/0.2 | 1000/85% |
| F-G/Sn@C [4] | 43.5 % | 413/0.1 | 100/98.3% |
| SnS_2_/RGO [5] | 29.2 % | 825/0.1 | 200/55% |
| a-SnO_2_/CNT [6] | 16 % | 630/0.1 | 300/69% |
| Sn_4_P_3_@C [7] | 19 % | 580/0.1 | 120/95% |
| RGO/SnS_2_@C [8] | 16.3 % | 651/0.1 | 200/ 89% |
| CNT/SnS_2_@C [8] | 26.4 % | 691/0.1 | 200/87.6% |
| \| a-Sn_2_P_2_O_7_/RGO [9] \|  \|  \|  \|  \|  \| \| --- \| --- \| --- \| --- \| --- \| --- \| | 15 % | 480/0.05 | 15000/70% |
| Sn@NCNFs [10] | 54 % | 600/0.1 | 1000/83% |
| G@C@SnO_2_ [11] | 37.9 % | 277.4/0.05 | 300/62% |
| SnO_2_/N,S-graphene [12] | 25.2 % | 392.9/0.05 | 120/99.2% |
| Sn/NMCs [13] | 48.2 % | 332/0.05 | 300/75.6% |
| SnS@SNCF-55 [14] | 44.4 % | 600/0.1 | 500/80% |
| Sn_4_P_3_@C [15] | 37 % | 420/0.2 | 4000/~85% |
| Sn@C [16] | 24 % | 822.5/0.169 | 300/80.1% |
| Sn_4_P_3_/RGO [17] | 10.4 % | 656/0.1 | 1500/~73% |
| SnP_2_O_7_/RGO [18] | 16.8 % | 213/0.2 | 1000/99% |
| 3D SnSb@N–PG [19] | 54.6 % | 400/0.1 | 4000/100% |
| SnO_2_/G-S [20] | 46 % | 650/0.1 | 90/77% |
| SnSe_2_@C [21] | 32.7 % | 360.1/0.5 | 1000/~75% |
| SnSe_2_/GNS [22] | 32.3 % | 598.9/0.2 | 4000/47.5% |
| Sn@SNC [23] | 58 % | 101.8/0.1 | 100/~67% |
| **This work** | **4.4 %** | **400/0.1** | **1200/92%** |

**Table S2. Comparison of cycling performance, rate capability and coulombic efficiency of different Na-DIBs (1 C = 100 mA g^-1^).**

| Anode/cathode configuration | Cycling performance  (cycles/capacity retention) | Rate capability (capacity retention) | Coulombic efficiency |
| --- | --- | --- | --- |
| Sn\|\|Graphite [24] | 400/94% | 78.2% at 5 C (relative to 1 C) | 92% at 1 C |
| FePO_4_\|\|Graphite [25] | 250/92.2% | Not shown | ~96% at 2 C |
| MoS_2_@C\|\|Graphite [26] | 200/85% | 53.8% at 10 C (relative to 2 C) | 90% at 2 C |
| TiO_2_\|\|Graphite [27] | 1400/70% | 82.9% at 15 C (relative to 0.5 C) | ~95% at 5 C |
| MoS_2_@NC\|\|Expanded graphite [28] | 500/61.5% | 48.9% at 20 C (relative to 2 C) | 90% at 10 C |
| Fe[Fe(CN)_6_]\|\|Graphite [29] | 400/~58% | 17.0% at 2.8 C (relative to 0.35 C) | 98.5% at 0.35 C |
| a-MoS_2_-NS@NSC_film_\|\|Graphite [30] | 300/88% | 57.0% at 10 C (relative to 1 C) | 93% at 1 C |
| Phosphorus-doped soft carbon\|\|Graphite [31] | 900/81.8% | 67.6% at 30 C (relative to 5 C) | 80% at 10 C |
| Hard carbon\|\|Graphite [32] | 200/92.4% | 63.9% at 5.6 C (relative to 0.37 C) | ~100% at 1 C |
| Soft carbon\|\|Graphite [33] | 800/81.8% | 40% at 20 C (relative to 2 C) | 90% at 5 C |
| Pine needle derived carbon\|\|KS_6_ graphite [34] | 1000/87.2% | 63.2% at 10 C (relative to 0.5 C) | 92.4% at 0.5 C |
| Soft carbon\|\|Expanded graphite [35] | 350/94% | 42.6% at 10 C (relative to 1 C) | ~97% at 1 C |
| Na\|\|Fe_2_(dobpdc) [36] | 50/90% | 72% at 2.8 C (relative to 0.07 C) | 99% at 0.7 C |
| Na_2_Ti_3_O_7_\|\|Coronene [37] | 5000/80% | 37.5% at 10 C (relative to 0.5 C) | 98% at 5 C |
| **This work** | **1000/96.3%** | **83.3% at 30 C (relative to 3 C)** | **98.3% at 3 C** |

**References**

1. Ma D, Li Y, Mi H*, et al.* Robust SnO_2-x_ nanoparticle-impregnated carbon nanofibers with outstanding electrochemical performance for advanced sodium-ion batteries. *Angew Chem Int Ed*. 2018; **57**(29): 8901-5.

2. Zheng Y, Zhou T, Zhang C*, et al.* Boosted charge transfer in SnS/SnO_2_ heterostructures: toward high rate capability for sodium-ion batteries. *Angew Chem Int Ed*. 2016; **55**(10): 3408-13.

3. Jiang Y, Wei M, Feng J*, et al.* Enhancing the cycling stability of Na-ion batteries by bonding SnS_2_ ultrafine nanocrystals on amino-functionalized graphene hybrid nanosheets. *Energy Environ Sci*. 2016; **9**(4): 1430-8.

4. Luo B, Qiu T, Ye D*, et al.* Tin nanoparticles encapsulated in graphene backboned carbonaceous foams as high-performance anodes for lithium-ion and sodium-ion storage. *Nano Energy*. 2016; **22**: 232-40.

5. Tu F, Xu X, Wang P*, et al.* A few-layer SnS_2_/reduced graphene oxide sandwich hybrid for efficient sodium storage. *J Phys Chem C*. 2017; **121**(6): 3261-9.

6. Cui J, Xu Z-L, Yao S*, et al.* Enhanced conversion reaction kinetics in low crystallinity SnO_2_/CNT anodes for Na-ion batteries. *J Mater Chem A*. 2016; **4**(28): 10964-73.

7. Fan X, Gao T, Luo C*, et al.* Superior reversible tin phosphide-carbon spheres for sodium ion battery anode. *Nano Energy*. 2017; **38**: 350-7.

8. Luo B, Hu Y, Zhu X*, et al.* Controllable growth of SnS_2_ nanostructures on nanocarbon surfaces for lithium-ion and sodium-ion storage with high rate capability. *J Mater Chem A*. 2018; **6**(4): 1462-72.

9. Yang X, Zhang R-Y, Zhao J*, et al.* Amorphous tin-based composite oxide: A high-rate and ultralong-life sodium-ion-storage material. *Adv Energy Mater*. 2018; **8**(8): 1701827.

10. Sha M, Zhang H, Nie YT*, et al.* Sn nanoparticles@nitrogen-doped carbon nanofiber composites as high-performance anodes for sodium-ion batteries. *J Mater Chem A*. 2017; **5**(13): 6277-83.

11. Wu Q, Shao Q, Li Q*, et al.* Dual carbon-confined SnO_2_ hollow nanospheres enabling high performance for the reversible storage of alkali metal ions. *ACS Appl Mater Interfaces*. 2018; **10**(18): 15642-51.

12. Wang H-G, Wu Q, Wang Y*, et al.* Molecular engineering of monodisperse SnO_2_ nanocrystals anchored on doped graphene with high-performance lithium/sodium-storage properties in half/full cells. *Adv Energy Mater*. 2019; **9**(3): 1802993.

13. Ying HJ, Zhang SL, Meng Z*, et al.* Ultrasmall Sn nanodots embedded inside N-doped carbon microcages as high-performance lithium and sodium ion battery anodes. *J Mater Chem A*. 2017; **5**(18): 8334-42.

14. Wang Y, Zhang Y, Shi J*, et al.* Tin sulfide nanoparticles embedded in sulfur and nitrogen dual-doped mesoporous carbon fibers as high-performance anodes with battery-capacitive sodium storage. *Energy Storage Mater*. 2019; **18**: 366-74.

15. Pan E, Jin Y, Zhao C*, et al.* Conformal hollow carbon sphere coated on Sn_4_P_3_ microspheres as high-rate and cycle-stable anode materials with superior sodium storage capability. *ACS Appl Energy Mater*. 2019; **2**(3): 1756-64.

16. Tian H, Liang Y, Repac J*, et al.* Rational design of core-shell-structured particles by a one-step and template-free process for high-performance lithium/sodium-ion batteries. *J Phys Chem C*. 2018; **122**(39): 22232-40.

17. Li Q, Li Z, Zhang Z*, et al.* Low-temperature solution-based phosphorization reaction route to Sn_4_P_3_/reduced graphene oxide nanohybrids as anodes for sodium ion batteries. *Adv Energy Mater*. 2016; **6**(15): 1600376.

18. Pan J, Chen S, Zhang D*, et al.* SnP_2_O_7_ covered carbon nanosheets as a long-life and high-rate anode material for sodium-ion batteries. *Adv Funct Mater*. 2018; **28**(43): 1804672.

19. Qin J, Wang T, Liu D*, et al.* A Top-down strategy toward SnSb in-plane nanoconfined 3D N-doped porous graphene composite microspheres for high performance na-ion battery anode. *Adv Mater*. 2018; **30**(9): 1704670.

20. Chen W, Song K, Mi L*, et al.* Synergistic effect induced ultrafine SnO_2_/graphene nanocomposite as an advanced lithium/sodium-ion batteries anode. *J Mater Chem A*. 2017; **5**(20): 10027-38.

21. Zhang F, Shen Y, Shao M*, et al.* SnSe_2_ Nanoparticles chemically embedded in a carbon shell for high-rate sodium-ion storage. *ACS Appl Mater Interfaces*. 2020; **12**(2): 2346-53.

22. Liu Y, Xu Y, Han Y*, et al.* Facile synthesis of SnSe_2_ nanoparticles supported on graphite nanosheets for improved sodium storage and hydrogen evolution. *Journal of Power Sources*. 2019; **436**: 226860.

23. Pan L, Huang H, Zhong M*, et al.* Hydrogel-derived foams of nitrogen-doped carbon loaded with Sn nanodots for high-mass-loading Na-ion storage. *Energy Storage Mater*. 2019; **16**: 519-26.

24. Sheng M, Zhang F, Ji B*, et al.* A novel tin-graphite dual-ion battery based on sodium-ion electrolyte with high energy density. *Adv Energy Mater*. 2017; **7**(7): 1601963.

25. Li C, Wang X, Li J*, et al.* FePO_4_ as an anode material to obtain high-performance sodium-based dual-ion batteries. *Chem Commun*. 2018; **54**(34): 4349-52.

26. Zhu H, Zhang F, Li J*, et al.* Penne-like MoS_2_/carbon nanocomposite as anode for sodium-ion-based dual-ion battery. *Small*. 2018; **14**(13): e1703951.

27. Wang X, Qi L,Wang H. Anatase TiO_2_ as a Na^+^-storage anode active material for dual-ion batteries. *ACS Appl Mater Interfaces*. 2019; **11**(33): 30453-9.

28. Li Z, Yang L, Xu G*, et al.* Hierarchical MoS_2_@N-doped carbon hollow spheres with enhanced performance in sodium dual-ion batteries. *ChemElectroChem*. 2018; **6**(3): 661-7.

29. Fan J, Fang Y, Xiao Q*, et al.* A Dual-ion battery with a ferric ferricyanide anode enabling reversible Na^+^ intercalation. *Energy Technol-Ger*. 2019; **7**(4): 1800978.

30. Liu Y, Hu X, Zhong G*, et al.* Layer-by-layer stacked nanohybrids of N,S-co-doped carbon film modified atomic MoS_2_ nanosheets for advanced sodium dual-ion batteries. *J Mater Chem A*. 2019; **7**(42): 24271-80.

31. Ma R, Fan L, Chen S*, et al.* Offset initial sodium loss to improve coulombic efficiency and stability of sodium dual-ion batteries. *ACS Appl Mater Interfaces*. 2018; **10**(18): 15751-9.

32. Hu Z, Liu Q, Zhang K*, et al.* All carbon dual ion batteries. *ACS Appl Mater Interfaces*. 2018; **10**(42): 35978-83.

33. Fan L, Liu Q, Chen S*, et al.* Soft carbon as anode for high-performance sodium-based dual ion full battery. *Adv Energy Mater*. 2017; **7**(14): 1602778.

34. Wang X, Zheng C, Qi L*, et al.* Carbon derived from pine needles as a Na^+^-storage electrode material in dual-ion batteries. *Global Challenges*. 2017; **1**(7): 1700055.

35. Yao X, Ke Y, Ren W*, et al.* Defect-rich soft carbon porous nanosheets for fast and high-capacity sodium-ion storage. *Adv Energy Mater*. 2018; **9**(6): 1803260.

36. Aubrey ML, Long JR. A dual-ion battery cathode via oxidative insertion of anions in a metal-organic framework. *J Am Chem Soc*. 2015; **137**(42): 13594-602.

37. Dong S, Li Z, Rodríguez-Pérez IA*, et al.* A novel coronene//Na_2_Ti_3_O_7_ dual-ion battery. *Nano Energy*. 2017; **40**: 233-9.
